# A Scene-Adaptive Trajectory Association Method for UAV Vision–AIS Applications in Airborne Maritime Perception

**DOI:** 10.3390/s26216790

**Published:** 2026-10-27

**Authors:** Yuanda Zhang, Yichen Tian, Chao Yuan, Kun Yu, Guang Yang, Haochen Si

**Affiliations:** 1 Aerospace Information Research Institute, Chinese Academy of Sciences, Beijing 100094, China; zhangyuanda24@mails.ucas.ac.cn (Y.Z.); tianyc@aircas.ac.cn (Y.T.); yukun@aircas.ac.cn (K.Y.); yangguang201924@aircas.ac.cn (G.Y.); sihaochen25@mails.ucas.ac.cn (H.S.); 2 University of Chinese Academy of Sciences, Beijing 100049, China

**Keywords:** Automatic Identification System, sensor data association, maritime perception, trajectory matching, UAV

## Abstract

**Highlights:**

**What are the main findings?**

- Propose a geospatial-temporal alignment mechanism for cross-domain data, addressing the challenge of “spatiotemporal inconsistency” in heterogeneous data through monocular geolocation and AIS trajectory reconstruction.
- Develop a scenario-driven dual-strategy association algorithm that adaptively switches between Instantaneous Synchronization Multi-feature Association (ISMA) and Spatiotemporal Constraint Weighted Dynamic Time Warping (STC-WDTW) based on scene complexity, overcoming the bottleneck of “inaccurate association” under complex sea conditions.

**What are the implications of the main findings?**

- The dual-strategy mechanism overcomes the limitations of traditional Euclidean distance matching and successfully addresses the key engineering challenges of fragmented drone trajectories and spatiotemporal asynchrony in multi-source data fusion.
- This framework establishes a data association foundation for cross-validation of multi-source data and analysis of potential maritime anomalies, offering potential applications in proactive maritime regulation.

**Abstract:**

Unmanned aerial vehicle (UAV) video and Automatic Identification System (AIS) data provide high-frequency visual observations and low-frequency identity/location messages, respectively. However, these two types of sensor data differ in spatiotemporal dimensions and pose engineering challenges for association and matching. This paper proposes a scene-adaptive trajectory association framework for airborne maritime perception. First, visual trajectories are mapped into a geospatial coordinate system using camera, gimbal, and flight platform metadata. Second, sparse AIS position sequences are filtered with physical constraints and locally interpolated to reconstruct continuous trajectories. Finally, based on target motion significance and local candidate complexity, the framework dynamically switches between Instantaneous Synchronization Multi-Feature Association (ISMA) and Spatiotemporal Constraint Weighted Dynamic Time Warping (STC-WDTW), leveraging kinematic features to deeply optimize the matching strategy, significantly enhancing robustness in trajectory association. This study collects data from the Port of Tianjin and adjacent coastal waters and evaluates the proposed method based on this dataset. Experimental results demonstrate that the proposed method achieves 94.34% IDP, 92.02% IDR, 93.17% IDF1, and 86.50% MOFA. The proposed method can be applied to marine dynamic monitoring systems, providing technical support and practical value for vessel traffic management in complex maritime areas and multi-source data fusion.

## 1. Introduction

Maritime traffic monitoring is increasingly relying on heterogeneous sensors, as no single modality can simultaneously meet the demands of maritime surveillance in terms of coverage, timeliness, mobility, and vessel attribute perception [1,2,3,4]. AIS can broadcast vessel identity, geodetic coordinates, speed, and heading, but its update intervals are irregular, and measurements may be missing, delayed, or even tampered with. Optical cameras provide continuous visual observation and high-resolution appearance information, yet they cannot directly deliver Maritime Mobile Service Identity (MMSIs), as defined by the International Telecommunication Union (ITU), or motion trajectories referenced to a global coordinate system. Drones extend optical sensing beyond fixed shore-based infrastructure, enabling rapid inspection of blind spots, anchorage areas, port entry/exit channels, and emergency incident sites [5,6,7,8]. Therefore, the integration of drone-AIS data is closely related to sensor system fusion, multimodal data processing, and intelligent perception in maritime environments.

This issue primarily involves two types of technical mismatches. First, drone video and AIS differ in both coordinate systems and sampling frequencies [9]. Video detectors operate at tens of frames per second in a pixel coordinate system, whereas AIS reports positions in a geodetic coordinate system at intervals ranging from seconds to minutes [10]; unlike fixed surveillance cameras, drone cameras continuously vary in pose, flight altitude, and gimbal orientation [11]. Second, the association becomes significantly more challenging when multiple vessels travel along similar routes, when visual geolocation exhibits systematic bias, or when short-term visual trajectories need to be compared with longer, sparsely sampled AIS trajectories [12]. Point-wise nearest-neighbor methods are prone to identity switching [13], while unconstrained dynamic time warping (DTW) may excessively align unrelated trajectories [14].

Although existing research has achieved certain success in fusing shore-based radar, shore-based CCTV, and AIS, significant limitations still emerge when these methods are directly applied to maritime monitoring from the high-dynamic perspective of drones. On the one hand, spatiotemporal consistency is a fundamental prerequisite for heterogeneous data fusion. Spatially, current studies generally assume that AIS broadcasts provide accurate position information, thereby unifying coordinates of multi-source data into the pixel domain, which results in the loss of critical information such as a ship’s actual geographic location, speed, and heading. Therefore, we need a method for reconstructing target geospatial trajectories from drone video. Temporally, AIS data and drone video inherently differ in sampling intervals and temporal granularity: AIS data updates infrequently and suffers from delays and packet losses, creating structural mismatches with the short-term continuous observations captured by drones. On the other hand, association accuracy determines the ultimate effectiveness of data fusion. Changes in drone attitude, camera viewpoints, and visual localization errors further cause spatial offsets and shape distortions between back-calculated trajectories and AIS-derived geospatial trajectories. Existing methods face challenges such as ambiguous associations among spatially proximate targets and unstable local motion characteristics under conditions of drone visual localization noise and dense multi-target scenes, lacking mechanisms to robustly associate targets using complete temporal trajectories and multidimensional kinematic features. Moreover, most current AIS fusion studies treat AIS as a reliable source of vessel identity and motion information without adequately addressing potential risks related to the authenticity of AIS data itself, such as anomalous positions or identity information reported by AIS [15].

In summary, the current research mainly has the following two core gaps:

1. Lack of robust video-AIS spatiotemporal alignment methods for high-dynamic drone platforms [16].
2. Existing trajectory association methods struggle to balance robustness under high-frequency localization noise and the physical interpretability required for maritime law enforcement [17].

To address the aforementioned research gap, this paper aims to propose a comprehensive algorithm for fusing heterogeneous data from drones and AIS by focusing on two aspects: “spatiotemporal alignment of visual targets and AIS trajectories” and “a dual-strategy trajectory association algorithm.”

1. Propose a method for extracting geospatial trajectories of targets from monocular drone video, while investigating short-term AIS trajectory reconstruction, achieving spatiotemporal alignment between drone vision and AIS in the geospatial domain.
2. Propose a scenario-driven dual-strategy matching framework combining drones and AIS, which integrates ship kinematic features to optimize traditional trajectory association methods without relying on AIS information beyond position, thereby improving association accuracy in maritime monitoring scenarios.

## 2. Related Work

### 2.1. Overview of Aerial and Maritime Multimodal Perception

Aerial and maritime perception is rapidly evolving from single-sensor modes toward a multimodal collaborative architecture [18]. Complementary sensing forms the physical foundation of this multimodal synergy. Current primary sources of maritime situational awareness include AIS, radar, visible-light cameras, infrared sensors, satellite SAR, and environmental data [19]. The complementarity among these modalities lies in their distinct capabilities: radar maintains sensitivity at long ranges, detecting echo signals before targets become visually identifiable [20]; electro-optical/infrared sensors provide fine-grained texture details, but distant targets collapse into few pixels, resulting in reduced discriminability; AIS delivers precise identity and location data, yet relies on cooperative targets and suffers from low update rates and significant asynchrony challenges. Multilayered, multimodal maritime surveillance systems integrate ground-based sensors, low-altitude UAVs, and satellite platforms. Case studies demonstrate that fused information effectively reduces false alarms and enhances geolocation accuracy of targets [21].

### 2.2. Visual-AIS Spatial and Temporal Alignment

Visual-AIS fusion can be achieved either by projecting AIS positions onto the image plane or by mapping visual detection results into a geographic coordinate system. For calibrated, fixed cameras with stable configurations, projection onto the image plane is more effective and has been applied in inland waterway monitoring [22]. However, projecting visual targets into a geographic coordinate system is more suitable when measuring distances, comparing trajectories, or integrating with other geospatial sensors. Aerial georegistration is more challenging due to time-varying camera centers, aircraft attitude, and gimbal orientation. Some studies have derived joint system error expressions using multi-platform observations to address target localization biases caused by unregistered raw data biases that may reach hundreds or even thousands of meters [23]. Others have employed weighted subspace fitting for maritime target positioning within integrated air-space-ground network environments [24]. Nevertheless, research indicates that monocular projection is highly sensitive to calibration errors, observation geometry, and scene elevation errors [25].

Time alignment is typically achieved through interpolation, resampling, or learned asynchronous matching [26,27]. The AMGT framework employs interpolation and linear prediction to synchronize AIS messages in time and reduces nonlinear misalignment caused by UAV motion via a motion-aware coordinate unification strategy [28]. DeepSORVF fuses AIS and video motion features for ship tracking and provides the FVessel public dataset for asynchronous maritime fusion [17]. Other recent systems combine ship detection, tracking, coordinate transformation, and DTW-based matching [29]; drone-oriented systems integrate visual and AIS information for ship identification and tracking [30]. These studies demonstrate the value of multimodal fusion, while also indicating that system performance largely depends on calibration accuracy, data synchronization quality, and scenario-specific association logic.

### 2.3. Heterogeneous Trajectory Association

Early research widely adopted classical association methods including nearest neighbor, global nearest neighbor, Hungarian assignment, joint probabilistic data association, and multiple hypothesis tracking [31,32,33]. In environments with dense targets or crossing trajectories, traditional matching approaches based purely on spatial distance struggle to reliably distinguish nearby targets, leading to association ambiguity and identity switching [34]. Recently, researchers have begun exploring the integration of multidimensional features such as position, velocity, and heading to enhance matching robustness [35,36]. Building upon this, trajectory-level methods reduce association ambiguity by comparing trajectory shapes [37,38,39]. DTW and longest common subsequence (LCSS) can accommodate sequences of unequal lengths and irregular sampling, but unconstrained alignment may produce physically implausible temporal distortions [40,41]. In recent years, fuzzy DTW, machine learning, and graph models or deep models have been applied to multi-source trajectory association [42,43,44]. The IEEE Sensors Journal published a radar-AIS deep learning association model that fuses trajectory and scene features [43]. The CA-MGNN method transforms AIS and radar trajectory points into multi-temporal-spatial dimensional features via an MGNN module, leveraging cross-attention mechanisms to build interactive features for association [44]. GMvA integrates AIS and CCTV data, capturing spatiotemporal characteristics of ship trajectories through temporal learning and graph neural networks [45]. The PGA-Track method addresses the asynchronous matching problem between AIS and video trajectories at the feature level using its spatiotemporally aware sequence Transformer encoder. Deep models can learn complex similarity functions but require representative paired data [46]. However, publicly available datasets pairing drone videos with AIS data remain extremely limited, particularly lacking strictly spatiotemporally synchronized annotations for airborne ship trajectory association. On the other hand, the results of visual-AIS association serve as critical foundations for subsequent tasks such as identifying abnormal vessels, analyzing target behaviors, and supporting maritime regulation. Although current deep learning models can be used for association decisions, they do not directly reveal physical evidence such as positional residuals, heading consistency, or temporal alignment quality, posing challenges in applications requiring explainable evidence, such as maritime law enforcement. The main findings and limitations of related and existing work are shown in Table 1.

## 3. Method

### 3.1. Perception Architecture and Problem Definition

The perception system receives four types of timestamped inputs: drone video, subtitle metadata containing camera and platform status, visual detection/tracking results, and raw AIS messages. Let the visual trajectory of the target be

(1) $$T_{v}^{V}={\left\{\left(p_{i}^{V},t_{i}^{V}\right)\right\}}_{i=1}^{n}$$

Reconstructed AIS trajectory of the candidate target *a* is

(2) $$T_{a}^{A}={\left\{\left(p_{j}^{A},t_{j}^{A}\right)\right\}}_{j=1}^{m}$$

The visual detection employs a UAV ship detection model based on YOLO-UAVShip [47], and continuous trajectories are formed by connecting it with BoT-SORT [48]. Instead of directly using ground speed or ground course from AIS messages as matching features, the association framework derives velocity and motion direction from reconstructed AIS position sequences, ensuring that the kinematic quantities involved in comparison originate from consistent physical sources while avoiding tampering with information other than position in AIS data. Finally, an adaptive dual-strategy trajectory association mechanism is adopted to achieve stable matching of heterogeneous data across different application scenarios.

### 3.2. Monocular Georeferencing of Drones

This paper constructs an imaging geometric model for drones based on the pinhole camera model and defines the five core spatial coordinate systems involved along with their geometric and physical meanings as follows:

1. Pixel coordinate system (o_uv_ − uv): The origin is located at the top-left corner of the digital image, with the horizontal axis as u and the vertical axis as v, measured in pixels.
2. Image physical coordinate system (o_xy_ − xy): The origin is located at the center of the image sensor’s optical axis, with the *x*-axis parallel to the u-axis pointing to the right and the *y*-axis parallel to the v-axis pointing downward, in units of millimeters (mm).
3. Camera coordinate system (O_c_ − X_c_Y_c_Z_c_): The origin O_c_ is located at the camera’s optical center. The Z_c_ axis points forward along the camera’s optical axis, while the X_c_ axis and Y_c_ axis are parallel to the *x*-axis and *y*-axis of the image physical coordinate system, respectively.
4. UAV body coordinate system (O_b_ − X_b_Y_b_Z_b_): The origin O_b_ is located at the center of mass of the UAV. A standard “forward-left-up” body frame is adopted, where the X_b_ axis points forward along the UAV’s nose, the Y_b_ axis points to the left side, and the Z_b_ axis is vertical upward relative to the body.(O_b_ − X_b_Y_b_Z_b_)O_b_X_b_Y_b_Z_b_.
5. East-North-Up (ENU) coordinate system (O_w_ − X_w_Y_w_Z_w_): The origin O_w_ is set at the drone’s takeoff position. The X_w_ axis points east (E), the Y_w_ axis points north (N), and the Z_w_ axis points upward, perpendicular to the reference ellipsoid of the Earth’s surface (U).

Let the position of the drone in the ENU coordinate system be $P_{u}={\left[X_{u},Y_{u},Z_{u}\right]}^{T}$ and let the midpoint of the bottom edge of the image object detection bounding box be **q** = [*u*,*v*,1]*^T^*. Then, its line-of-sight vector in the ENU coordinate system is

(3) $$r^{w}=R_{b}^{w}R_{c}^{b}K^{-1}q$$

Here, **K** is the camera intrinsic matrix, $R_{c}^{b}$ represents the rotation from the camera coordinate system to the body coordinate system, and $R_{b}^{w}$ represents the rotation from the body coordinate system to the ENU coordinate system.

Assuming that the selected image positioning anchor is located on the local sea surface plane *Z* = *Z_s_*, the intersection point of the ray with the plane has a scale of

(4) $$\lambda =\frac{Z_{s}-Z_{u}}{r_{z}^{w}},\;r_{z}^{w}<0$$

The horizontal position of the target is

(5) $$\left[\begin{array}{c} X_{t}\\ Y_{t} \end{array}\right]=\left[\begin{array}{c} X_{u}\\ Y_{u} \end{array}\right]+\lambda \left[\begin{array}{c} r_{x}^{w}\\ r_{y}^{w} \end{array}\right]$$

Then convert the ENU coordinates to WGS-84 latitude and longitude.

Apply a constant-velocity Kalman filter to the visual trajectory [49]. Let the state be $x_{k}={\left[E_{k},N_{k},v_{k}^{E},v_{k}^{N}\right]}^{T}$ and the state transition matrix corresponding to the time interval Δ*t* be

(6) $$F_{k}=\left[\begin{array}{cccc} 1 & 0 & \Delta t & 0\\ 0 & 1 & 0 & \Delta t\\ 0 & 0 & 1 & 0\\ 0 & 0 & 0 & 1 \end{array}\right]$$

**Figure 1:**
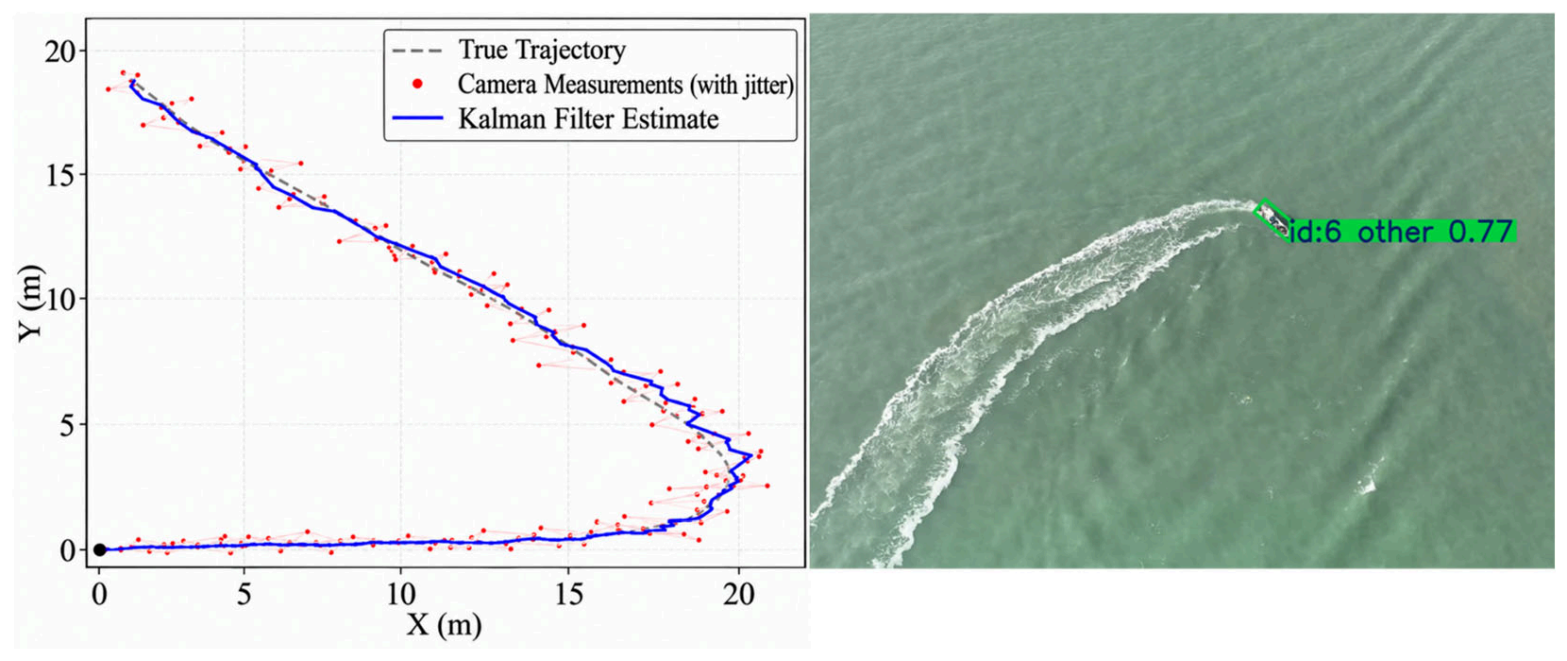
Kalman filter effect.

To address the acceleration and deceleration phenomena observed in certain small, highly maneuverable vessels, the filter compensates for model truncation errors by introducing a process noise covariance matrix, thereby enabling the model to adaptively tolerate unknown accelerations. The performance of the Kalman filter is illustrated in Figure 1.

### 3.3. AIS Data Filtering and Trajectory Reconstruction

AIS is limited by VHF line-of-sight propagation, multipath interference, and message collisions in high-density maritime areas. As a result, raw AIS data not only has a low sampling frequency but also often contains numerous duplicate messages and significant anomalous drift points, along with severe data truncation issues. To match the high-frequency drone visual frames, it is necessary to deduplicate and clean the raw AIS data and reconstruct continuous vessel AIS trajectories using interpolation algorithms.

To distinguish offline interpolation evaluation from real-time association, two processing modes are adopted. In the offline interpolation experiments (Section 4.4), the complete cleaned AIS trajectories, including past and future observations, are used solely to compare the reconstruction accuracy of different interpolation methods. In contrast, during causal association, only AIS messages received and available at the current evaluation time are used. No future AIS messages are accessed for trajectory reconstruction or matching, and the reconstructed AIS trajectory is updated as new messages arrive.

This paper compares four interpolation methods: linear interpolation, piecewise cubic Hermite interpolation (PCHIP) [50], cubic spline interpolation [51], and Akima interpolation [52]. The reconstructed trajectory is resampled according to the time resolution required by the association method. Akima interpolation has local characteristics, which can reduce the overshooting that may occur with globally smoothed cubic splines during ship turns or near-stationary conditions.

### 3.4. Scene-Adaptive Strategy Selection

This study reduces the localization error of drone visual back-calculated trajectories by introducing multidimensional kinematic features and an improved dynamic time warping algorithm from a global temporal perspective. However, a single association rule is not suitable for all maritime scenarios. For stationary or low-speed vessels, due to the positioning jitter inherent in monocular vision, the back-calculated geographic trajectory degrades into “unordered random scatter points” near the origin. In such cases, the instantaneous velocity and heading angle extracted from coordinate differences undergo severe oscillations, resulting in meaningless pseudo-motion features. Moreover, in sparse scenes, synchronous comparison with relatively low computational cost is sufficient. Conversely, sequence-level reasoning is only required when multiple moving vessels exist within the same candidate gate.

To achieve precise strategy triggering, the system defines two core evaluation operators, which are $T_{v}^{V}$, for the current visual tracking target sequence within each time domain sliding window.

The motion saliency operator is used to distinguish whether a target’s trajectory represents genuine physical displacement or error caused by visual localization noise. It is defined as the ratio of the target’s displacement within a time window ΔT to the actual accumulated path length of the trajectory points.

(7) $$M\left(T_{v}^{V}\right)=\frac{\mathrm{Dist}\left(P_{v,t},P_{v,t-\Delta T}\right)}{\sum_{k=t-\Delta T+1}^{t}\mathrm{Dist}\left(P_{v,k},P_{v,k-1}\right)+\epsilon }$$

Here, *ϵ* is a small constant introduced to prevent division by zero. When the vessel is actually moving, the trajectory exhibits strong directionality and ℳ(T_v_) approaches 1; when the vessel is stationary or idling, positioning errors cause the trajectory to display Brownian-like random fluctuations, resulting in a cumulative path much greater than the net displacement, with the ℳ(T_v_) decay approaching 0. The operator pseudocode is presented in Algorithm 1.
**Algorithm 1:** Motion Saliency Calculation**Input:** P—Target position; ΔT—time window; *ε*—small constant **Output:** ℳ—Motion saliency value 1  net_dist ← Distance from P(v, t) to P(v, t − ΔT); 2  cum_path ← 0; 3  **for** k **from** t − ΔT + 1 **to** t **do** 4      cum_path ← cum_path + Distance from P(v, k) to P(v, k − 1); 5    ℳ ← net_dist/(cum_path + *ε*); 6  **return** ℳ

The local scene complexity operator is used to assess the congestion level of the surrounding waters and the potential risk of ambiguity. It is defined as the number of other targets within a neighborhood *v* centered on the target with a given radius R_gate_:

(8) $$C\left(T_{v}^{V}\right)=\sum_{q\neq v}I\left({∥{\hat{p}}_{v}^{V}-{\hat{p}}_{q}^{V}∥}_{2}\leq R_{g}\right)$$

where $\hat{p}$ represents the current filtering position. When C(T_v_) ≥ 1, this indicates that multiple vessels are closely approaching in parallel or crossing paths, making single-point spatial distance highly prone to identity mismatches. The operator pseudocode is presented in Algorithm 2.
**Algorithm 2:** Local Scene Complexity Calculation**Input:** V—Target, Q—other targets, R—neighborhood radius **Output:** C—Scene complexity value1  C ← 0 2  **for** each target q in other targets Q **do** 3        **if** q ≠ V **then** 4                  spatial_dist ←Distance from P_V_ to P_q_ 5                        **if** spatial_dist ≤ R **then** 6                                C ← C + 1 7  **return** C

ℳ_th_ is set as the motion significance threshold; for any given drone video frame, the local scene complexity operator is computed for each target in the current frame and the motion salience operator within the time window. When the system meets any of the following conditions, the target is assigned to the ISMA algorithm for matching:

- Condition A: ℳ(T_v_)<ℳ_th_ indicates that the cumulative path of the vessel differs significantly from its actual displacement, suggesting that the vessel is nearly stationary or moving at a very low speed. The reconstructed motion characteristics are highly likely to be “false features” caused by positioning noise.
- Condition B: ℳ(T_v_) ≥ ℳ_th_ and C(T_v_) = 0, indicating that although the ship is moving at a certain speed, there are no other ships within its radius s R_gate_, resulting in a relatively low association difficulty.

The STC-WDTW algorithm is triggered if and only if the system simultaneously satisfies both effective motion characteristics and complex scene conditions.

- Condition C: ℳ(T_v_) ≥ ℳ_th_ and C(T_v_) ≥ 1 indicate a high ship density with all vessels in motion, making association difficult and prone to ambiguity; thus, the high-dynamic anti-ambiguity mode is activated.

**Figure 2:**
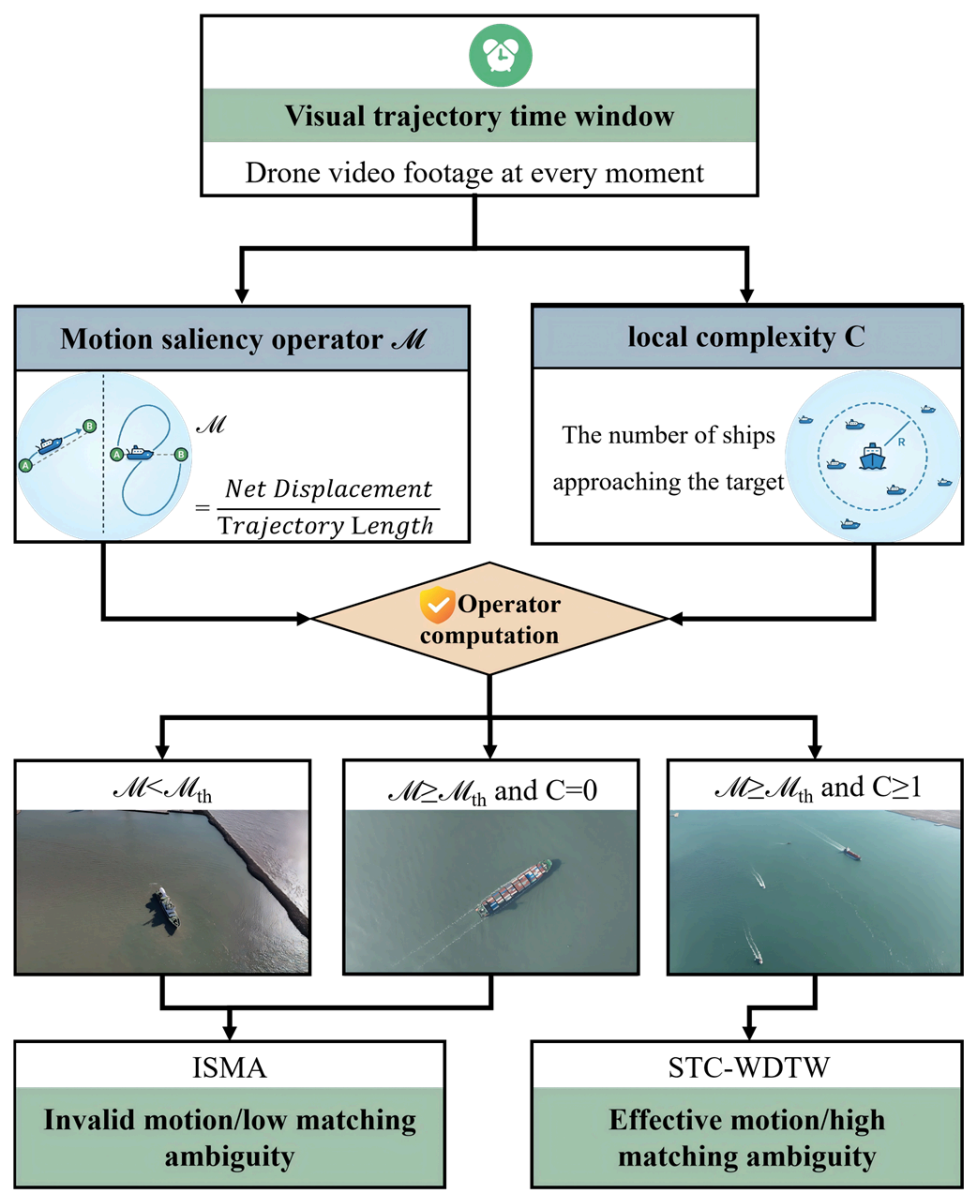
Scenario-Driven Strategy Selection Mechanism.

The complete strategy selection mechanism is illustrated in Figure 2.

### 3.5. Instantaneous Synchronization of Multi-Feature Association

**Figure 3:**
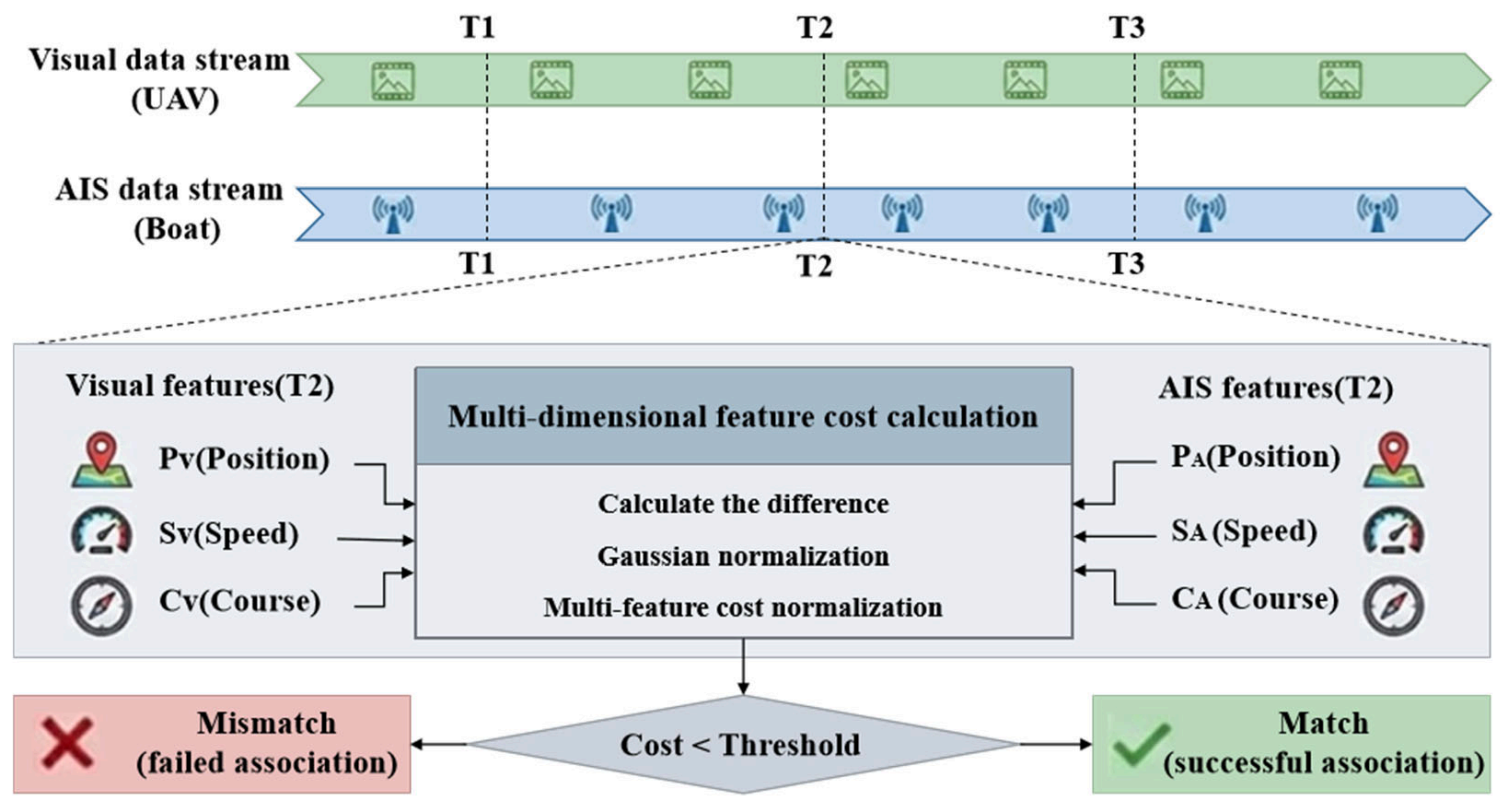
Schematic diagram of the ISMA algorithm principle.

ISMA searches for the temporally closest AIS reconstruction point for each visual feature; when this time difference $|t_{i}^{V}-t_{j}^{A}|>\Delta t_{\max}$ (Δ*t*_max_ in this paper is set to 3 s), the corresponding visual frame is directly considered a failed match in the ISMA computation branch, with its matching cost set to infinity and excluded from subsequent feature calculations. The complete ISMA algorithm workflow is illustrated in Figure 3.

At the feature matching level, to overcome the vulnerability of single spatial Euclidean distance measurement, this paper defines the local matching cost between a visual trajectory ${\left\{p_{i}^{V}\right\}}_{i=1}^{n}$ and an AIS trajectory ${\left\{p_{j}^{A}\right\}}_{j=1}^{m}$ as a composite distance based on multiple features:

Set d_pos_(i,j) as positional proximity, used to describe spatial closeness:

(9) $$d_{\mathrm{pos}}(i,j)=\left\|p_{i}^{V}-p_{j}^{A}\right\|$$

d_vel_(i,j) is the speed difference, reflecting the motion consistency between the two trajectories:

(10) $$d_{\mathrm{vel}}(i,j)=\left|\left\|v_{i}^{V}\right\|-\left\|v_{j}^{A}\right\|\right|$$

where $v_{i}^{V}$ and $v_{j}^{A}$ represent the velocities of the visual trajectory and AIS trajectory at that moment, respectively.

d_course_(i,j) is the heading difference, used to describe the consistency of the movement direction:

(11) $$d_{\mathrm{course}}(i,j)=\min(\left|c_{i}^{V}-c_{j}^{A}\right|,360{}^{\circ}-\left|c_{i}^{V}-c_{j}^{A}\right|)$$

where $c_{i}^{V}$ and $c_{j}^{A}$ represent the headings of the visual trajectory and AIS trajectory at that moment, respectively.

To achieve the fusion of association costs for different kinematic features on a unified scale, this paper first employs a Gaussian-type exponential function to map the residuals of each feature into a bounded cost space [53]:

(12) $$c_{\theta }(i,j)=1-\exp\left(-\frac{d_{\theta }^{2}(i,j)}{2\sigma_{\theta }^{2}}\right),\;\theta \in \left\{\mathrm{pos},\;\mathrm{vel},\;\mathrm{course}\right\}$$

To overcome systematic errors, noise, and local mismatches caused by AIS anomalies, this study introduces corresponding weight functions for three types of features to achieve a more robust matching process. The spatial consistency weight *α_p_*(i,j) suppresses the excessive influence of point pairs with large spatial distances on DTW matching results; the velocity consistency weight *α_v_*(i,j) highlights point pairs with inconsistent velocity vectors, effectively identifying delayed AIS updates and improving matching accuracy when positional information is subject to systematic errors; the heading consistency weight *α_c_*(i,j) supplements vessel motion state information, emphasizing differences in maritime vessel movement directions. When vessels are nearly stationary and motion characteristics become ineffective, both w_k_ = 0 and w_t_ = 0, and the algorithm degrades to a global nearest-neighbor approach based solely on position.

Based on this, a weighted normalized quadratic form composite cost is constructed using the normalized weights $\alpha_{\theta }(\sum_{\theta }\alpha_{\theta }=1)$, and the final association cost is obtained through an exponential mapping identical to that used for single features [54,55].

(13) $$D^{2}(i,j)=\sum_{\theta }\alpha_{\theta }{\left(\frac{d_{\theta }(i,j)}{\sigma_{\theta }}\right)}^{2}$$

(14) $$C(i,j)=1-\exp\left(-\frac{D^{2}(i,j)}{2}\right)$$

The final composite cost is expressed as:

(15) $$C(i,j)=1-\prod_{i}{\left(1-c_{\theta }(i,j)\right)}^{\alpha_{\theta }}$$

The composite cost lies within [0, 1) and increases monotonically with the weighted quadratic form. This construction draws inspiration from the exponential form of Gaussian radial basis functions, aiming to unify the dimensions and cost scales across different features and provide a continuous, bounded association cost for integrated ranking of multidimensional features. Any feature mismatch *c_θ_* → 1 results in *C* → 1, meaning that each feature possesses veto power when experiencing extreme mismatches. The final trajectory-level cost is obtained as the average cost over valid point pairs.

### 3.6. Multidimensional Feature Weighted Dynamic Time Warping

**Figure 4:**
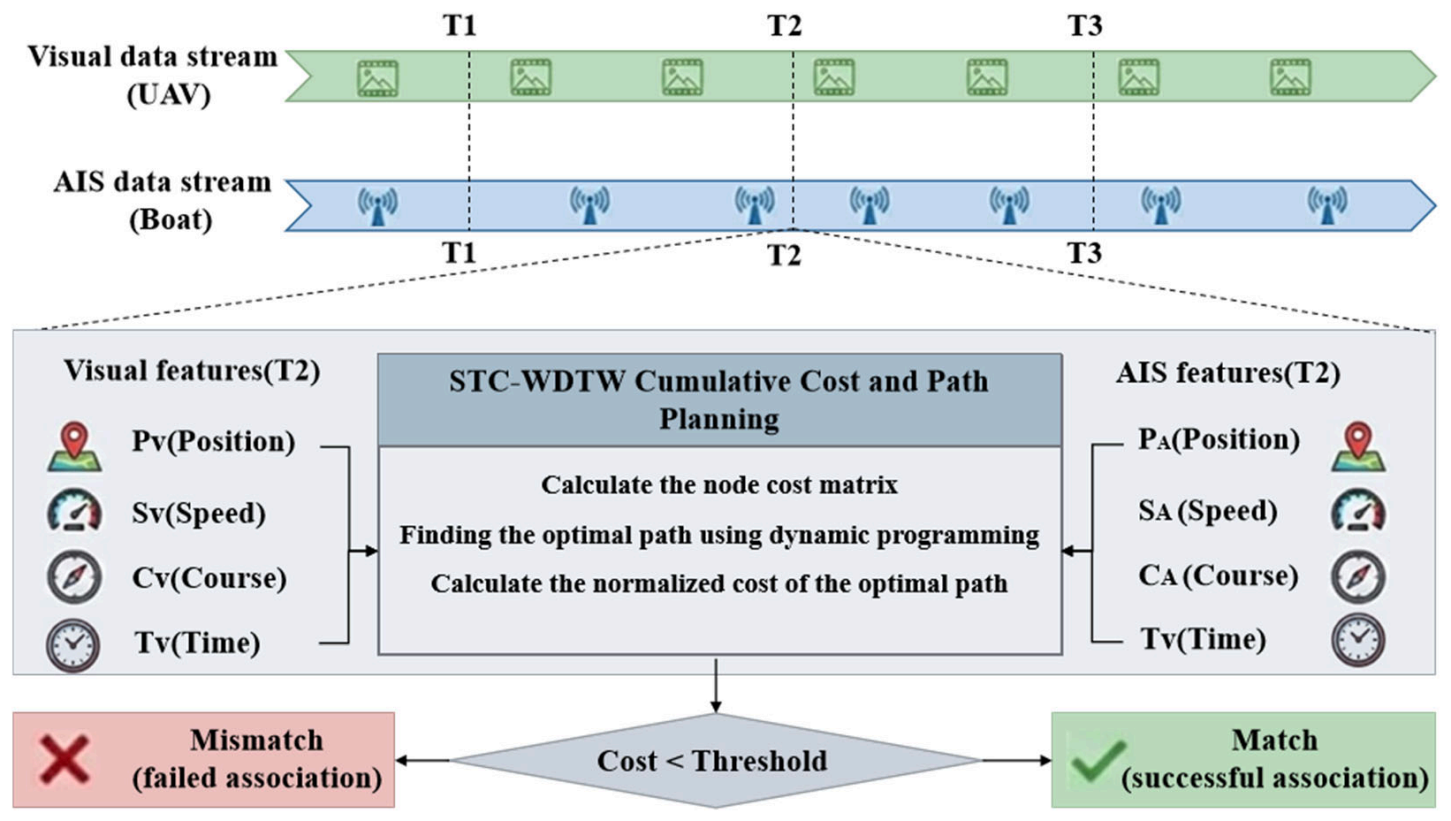
Schematic diagram of the STC-WDTW algorithm principle.

As maritime monitoring scenarios become increasingly complex, with vessel distributions shifting from sparse to dense and ships no longer moving in simple, stable patterns but instead engaging in parallel navigation, intersections, and evasive maneuvers, combined with intermittent AIS data outages, the performance of ISMA algorithms rapidly deteriorates. This paper conducts a deep spatiotemporal and kinematic enhancement of the traditional DTW algorithm, proposing the STC-WDTW algorithm. The complete STC-WDTW algorithm workflow is illustrated in Figure 4. Its core improvements are reflected in the following three dimensions:

1. Multidimensional kinematic weighting

Breaking the limitation of traditional DTW, which relies solely on spatial Euclidean distance to construct the cost matrix, STC-WDTW inherits the multidimensional feature fusion idea from previous work and incorporates physical-derived features such as position, velocity, and heading into the joint cost calculation. This upgrades trajectory alignment from a purely geometric comparison to a high-confidence verification of vessel motion states.

2. Open Start/End Subsequence Optimization

To address the issue of spatiotemporal misalignment caused by short visual trajectories or sudden AIS signal interruptions, this paper abandons the strict boundary condition of traditional DTW that requires forced alignment at both endpoints. This strategy allows shorter drone visual trajectories to be treated as subsequences, sliding and optimizing within a longer local AIS historical trajectory window, thereby avoiding matching deadlocks caused by partial data loss.

3. Adaptive Time Penalty Mechanism

To limit unbounded timeline distortions, this paper innovatively introduces a fifth-dimensional feature—time difference—into the multidimensional cost function.

(16) $$d_{\mathrm{time}}(i,j)=\left|T_{v_{i}}-T_{a_{j}}\right|$$

Set w_time_ as the time penalty weight, and locally calculate the normalized composite cost using Formulas (13)–(16):

(17) $$C_{\mathrm{node}}(i,j)=1-\prod_{i}{\left(1-c_{\theta }(i,j)\right)}^{\alpha_{\theta }},\theta \in \left\{\mathrm{pos},\;\mathrm{vel},\;\mathrm{course},\;\mathrm{time}\right\}$$

STC-WDTW aligns short visual sequences with candidate AIS trajectory segments. This method employs open-endpoint processing, allowing visual sub-trajectories to be matched without being forced to align with both endpoints of longer AIS sequences. After introducing the aforementioned multi-feature cost, the DTW accumulated cost matrix C(i,j) is defined as:

A(i, j) = *C*_node_(i, j) + min{A(i − 1, j), A(i, j − 1), A(i − 1, j − 1)} (18)

To quickly identify candidate AIS signals, the allowable matching area is constrained by the following equation:

(19) $$\Omega =\{\left(i,j\right):\left|t_{i}^{V}-t_{j}^{A}\right|\leq T_{w}\}$$

where T_windows_ is the time window, which is set to 3 s in this paper. For nodes not within the window, their cost is forcibly set to infinity. Meanwhile, the video frame rate is reduced to 1 Hz to accommodate the asynchronous sampling characteristics between the UAV’s high frame rate and AIS’s low update frequency, thereby avoiding unreasonable temporal alignment paths.

To meet real-time vessel monitoring requirements, the formal deployment mode only allows $t_{j}^{A}\leq t_{i}^{V}$, with $0\leq t_{i}^{V}-t_{j}^{A}\leq T_{w}$. Before sequence alignment, visual trajectories are resampled to 1 Hz to match the interpolated and reconstructed AIS frequency. The final spatiotemporal similarity score is defined as the ratio of the optimal path’s accumulated cost to its path length. The association is performed causally at each evaluation time, without intentional look-ahead or waiting for future AIS messages. The 3-s constraint limits the allowable time difference between matched observations, but this does not represent additional processing delay. Experiments show that the proposed matching algorithm achieves a processing rate of approximately 140 frames per second, although end-to-end latency from target detection has not been separately quantified.

### 3.7. Parameter Settings

1. Adaptive strategy parameters

Since the AIS dynamic reporting interval for ships in navigation is approximately 3 s, and most vessels can be approximated as moving at a constant velocity in a straight line within this interval, setting the time window of the motion significance operator ΔT to 3 s preserves the true direction while preventing positioning noise from overwhelming the net displacement due to excessively short movement intervals. Based on this, with a drone video frame rate of 30 fps, the theoretical ratio of two-dimensional random walk for completely stationary ships is about 0.105; thus, the motion significance threshold ℳ_th_ is set to 0.5, serving as a conservative midpoint between “random jitter” and “directed motion.” Considering monocular drone video localization errors, AIS signal positioning errors, and ship width, the local scene complexity operator R_gate_ is set to 500 m.

2. Feature normalization parameters

As shown in Table 2, the Gaussian bandwidths for each feature are initialized based on the preliminary measured error scale and temporal correlation constraints under current experimental conditions:

The above parameters are used to normalize the scales of different kinematic features and adjust the influence degree of each feature’s residual on the normalized association cost and do not directly correspond to the statistical standard deviation of sensor noise or the matching confidence threshold. Based on this, with a fixed initial bandwidth, subsequent weight optimization experiments are conducted to study the relative contributions of different features to trajectory association performance.

The final matching threshold is selected based on the training set. For formula 15, set the acceptable root mean square of weighted standardized residuals D*_th_*. Thus, the value of the composite cost threshold C*_th_* is related to D*_th_*, as shown in Table 3.

## 4. Experiments

### 4.1. Study Area, Sensors, and Dataset

**Figure 5:**
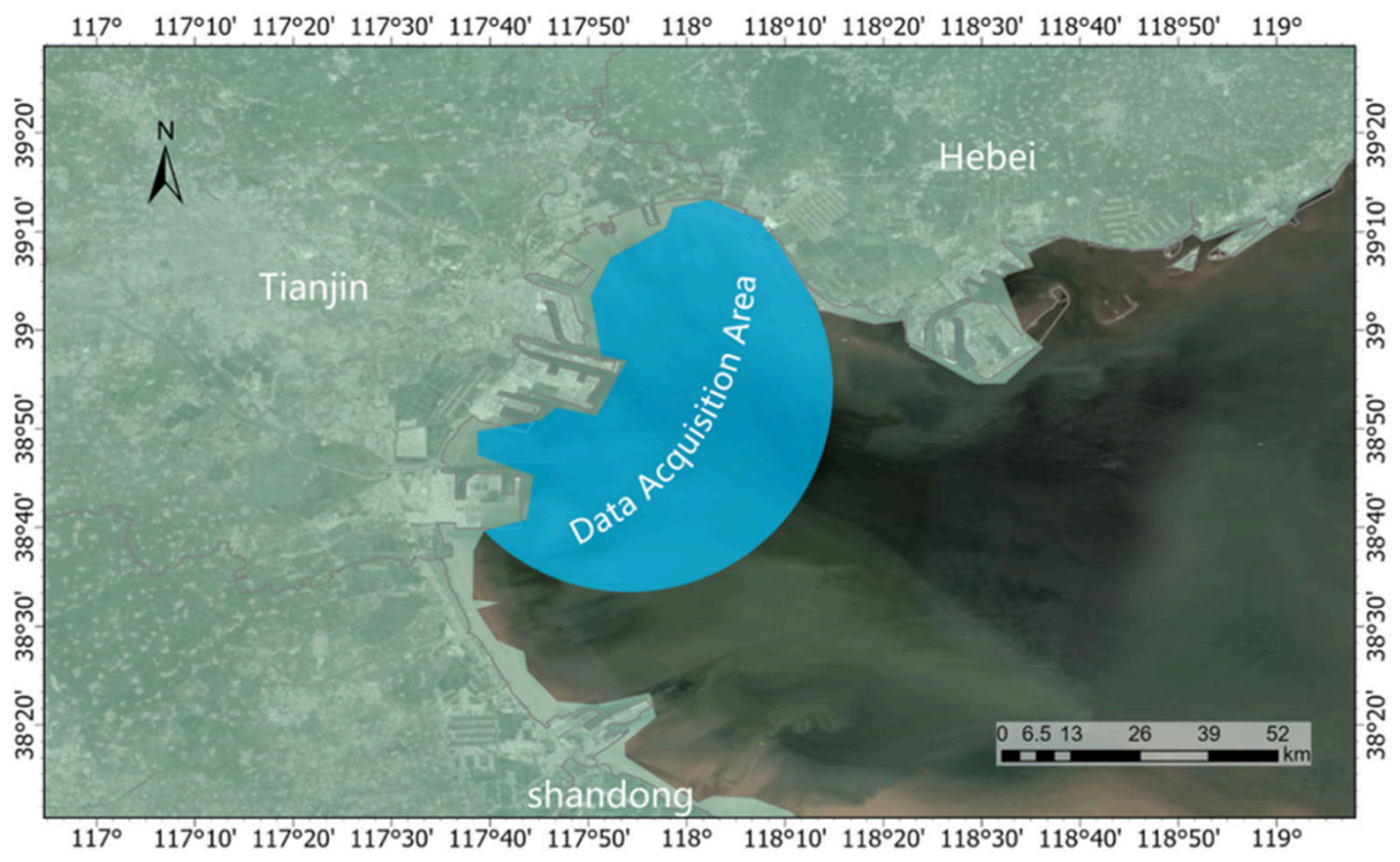
Data Collection Area. Two circles with a radius of 35 km are constructed through the two intersection points of the boundary of Tianjin Binhai New Area with the sea surface, and the area enclosed by these circles and the land boundary constitutes the data collection region of this study.

Field data was collected from the port of Tianjin and adjacent coastal waters to the east, as shown in Figure 5. DJI M3E and M4E multi-rotor drones were used as mobile optical sensing platforms. Their subtitle metadata files provide timestamps, focal length information, aircraft position and attitude, gimbal orientation, and flight altitude corresponding to video frames. AIS messages were received by HS-1206 and HS-6206GT devices mounted on the drones, connected to omnidirectional antennas, and transmitted in real time via a digital communication link back to a local program for parsing.

Table 4 summarizes the dataset. The data collection scheme covers flight altitude, different gimbal pitch angles, vessel density and navigation status, collection locations, collection time, and environmental lighting conditions (including strong backlighting, large-scale water surface reflection, and shadows).

### 4.2. Truth Value Construction

**Figure 6:**
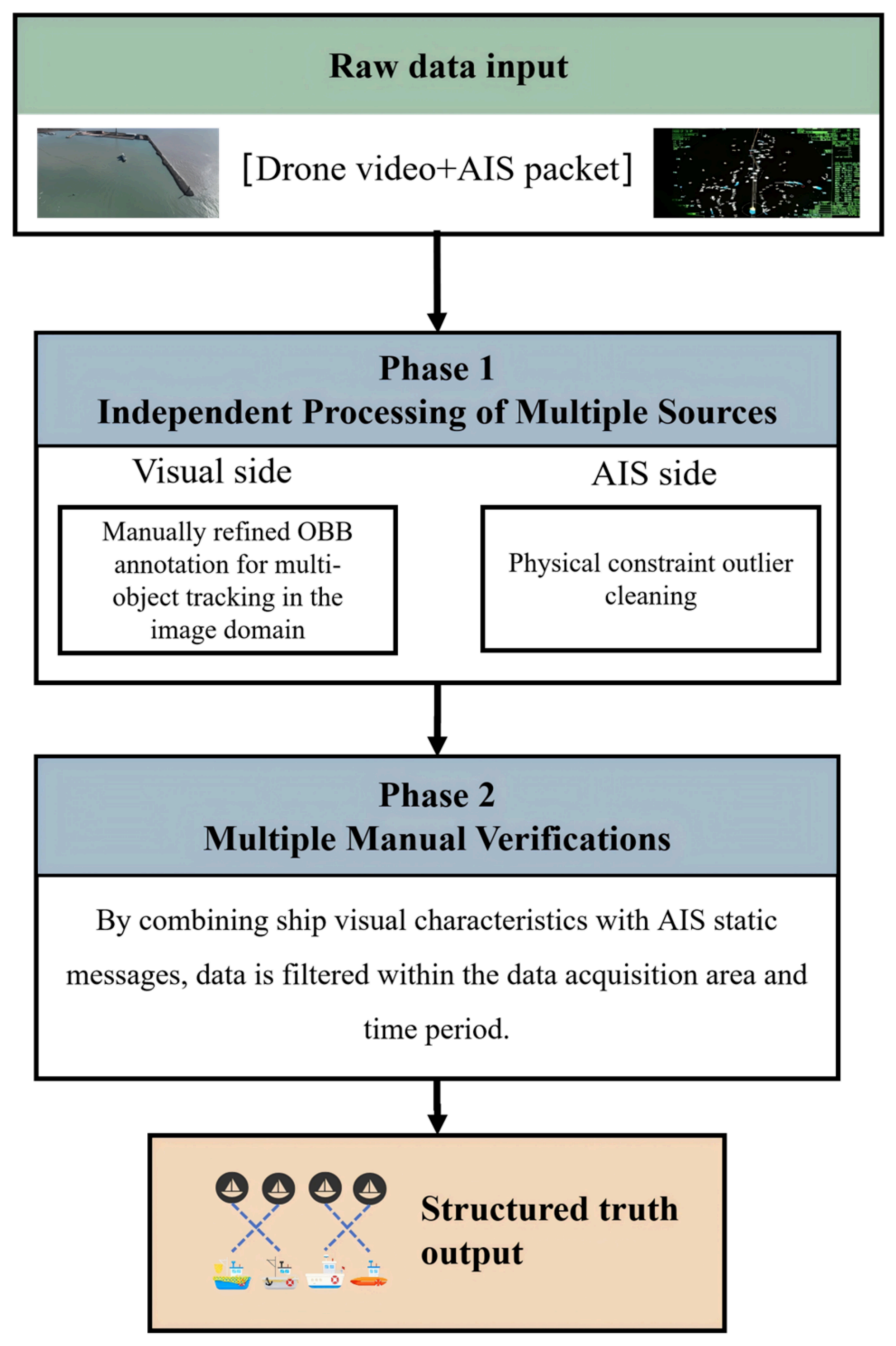
Truth value construction process used in this experiment.

The dataset construction process is shown in Figure 6. First, for visual trajectory extraction, we use professional annotation tools to sample and label vessels in drone video streams and then input the pre-trained vessel tracking model for retraining to extract continuous vessel tracking IDs and rotated bounding boxes (OBBs). Meanwhile, kinematic constraints such as maximum speed, turning rate, and acceleration are introduced into the cleaned raw AIS data to eliminate positioning drift points. Subsequently, within the corresponding time and spatial regions of the video, we manually verify the visual IDs against MMSIs by cross-referencing vessel features, including paint schemes, aspect ratios, superstructures, names, and static AIS fields. Finally, a structured ground truth matrix is generated containing [video frame number, visual target ID, image OBB, true MMSI/non-cooperative target label]. Finally, to explore the optimal weight combination of the proposed method on this dataset and conduct ablation studies, we initially screen the original 146 video clips, removing those used for geolocation accuracy testing, as well as clips shorter than 30 s or with poor video quality, retaining 136 valid videos. These are then randomly split into training and test sets in a 1:1 ratio, with each subset comprising 68 drone video clips. To verify the independence of the two subsets, we checked the unique vessel identities (MMSIs) and video identifiers in the training and test sets. The training set contains 177 vessels across 68 video clips, whereas the test set contains 163 vessels across 68 clips. No vessel identities or video recording sessions overlap between the two sets. This separation reduces the risk of information leakage caused by repeated observations of the same vessel or recording session.

To quantitatively evaluate the accuracy of visual target trajectory extraction in this study, a small fishing boat approximately 6 m long was selected. A professional RTK device with centimeter-level positioning error was installed on board, and the vessel maintained AIS signal transmission. According to the experimental design, a drone equipped with an AIS signal receiver collected data at various altitudes, angles, distances, and monitoring scenarios, ultimately obtaining real vessel trajectory data corresponding to the ship’s AIS signals.

Association performance can be evaluated using IDP, IDR, IDF1 and MOFA [17,56,57]:

(20) $$\mathrm{IDP}=\frac{\mathrm{TP}}{\mathrm{TP}+\mathrm{FP}}$$

(21) $$\mathrm{IDR}=\frac{\mathrm{TP}}{\mathrm{TP}+\mathrm{FN}}$$

(22) $$\mathrm{ID}F_{1}=\frac{2\mathrm{PR}}{P+R}$$

(23) $$\mathrm{MOFA}=1-\frac{\mathrm{FN}+\mathrm{FP}}{\mathrm{GT}}$$

where TP, FP, FN and GT are the numbers of the MMSI true positives, MMSI false positives, MMSI false negatives, and MMSI ground truth.

### 4.3. Monocular Geodetic Coordinate Inversion Results

To quantitatively validate the positioning accuracy and robustness of the monocular vision-based drone geocoordinate back-calculation model under real sea conditions, this section designs and tests three typical maritime monitoring experimental scenarios. The experiments use mean absolute error (Mean) as the primary evaluation metric, with minimum error (Min) and maximum error (Max) serving as supplementary criteria to precisely quantify the physical spatial deviation after converting the back-calculated latitude and longitude into geodetic coordinates. Based on the combined motion states of the drone and the target vessel, this study classifies the experiments into the following three scenarios:

1. “Ship moving, aircraft flying” dual dynamic scene

This scenario represents the most complex dual-dynamic condition closely resembling actual maritime patrol supervision. During the experiment, the target vessel traveled across the sea at a conventional speed of 8 to 15 knots, while the drone moved in the same direction as the ship. Under these conditions, the drone’s three-dimensional geospatial position, flight velocity, and gimbal attitude angles were continuously and dramatically adjusting, further complicated by micro-frequency vibrations caused by high-altitude wind fields over the ocean. As shown in Table 5, experimental results showed that the reverse-calculated pixel points exhibited significant divergence and sudden jumps.

2. “Ship moves, airplane remains still” scenario

This scenario primarily simulates a drone performing fixed-point hovering surveillance or monitoring tasks over a specific sea area. During the experiment, the drone hovers at approximately constant altitude and geographic position, while the target vessel navigates uniformly within the field of view, performs large-angle turns, or changes course to avoid collisions. Under these conditions, system errors mainly arise from slight vibrations of the gimbal caused by airflow disturbances during hover, as well as perspective projection distortions at image edges. In terms of positioning performance, as shown in Table 6, as the vessel moves from the distant background into the foreground of the frame, or approaches from the edge of the digital image toward the central principal point region, the reverse calculation accuracy shows a consistent increasing trend.

3. “The ship doesn’t move, but the plane takes off” scenario

This scenario primarily focuses on monitoring stationary targets (such as illegally anchored vessels or vessels berthed without signals) in maritime supervision. During the experiment, the target vessel remains completely stationary or exhibits slight motion due to waves, while the drone performs linear cruising, diving, or large-radius circling flights for imaging. Under these conditions, the pixel coordinates of the vessel in the digital image plane change entirely due to the drone’s relative motion and significant changes in viewing angle. As shown in Table 7, when the UAV flew at high speed or executed sharp turns, microsecond-level latency in sensor timestamp synchronization caused the latitude and longitude values derived through inverse computation to exhibit a disordered annular or scattered “spurious motion pattern” around the actual stationary positions of the vessels.

### 4.4. AIS Trajectory Reconstruction

To evaluate the trajectory reconstruction capabilities of different interpolation algorithms when dealing with real AIS data at various frequencies, this study uses cleaned AIS trajectories as approximate ground truth and conducts comparative experiments on four interpolation methods: Linear, Cubic, Pchip, and Akima. Algorithm performance is assessed from two aspects—position error and overall trajectory similarity—and further analysis is performed on accuracy variations under different sampling intervals. This experiment is an offline interpolation benchmark rather than a causal association evaluation. Complete cleaned AIS trajectories are used to establish reference values and compare interpolation methods; future observations used in this benchmark are not available to the real-time association module described in Section 3.3, Section 3.4, Section 3.5 and Section 3.6.

The experiment first groups AIS data by MMSI and segments trajectories based on time intervals. To avoid interference from abnormal AIS points affecting evaluation results, this study performs data cleaning to remove position outliers and clearly anomalous jump points. Subsequently, each trajectory is downsampled at six intervals—10 s, 30 s, 60 s, 120 s, 300 s, and 600 s—to simulate different AIS reporting frequencies. After downsampling, interpolation is performed using the Linear, Cubic, Pchip, and Akima methods, and the interpolated results are evaluated against true values for error analysis.

**Figure 7:**
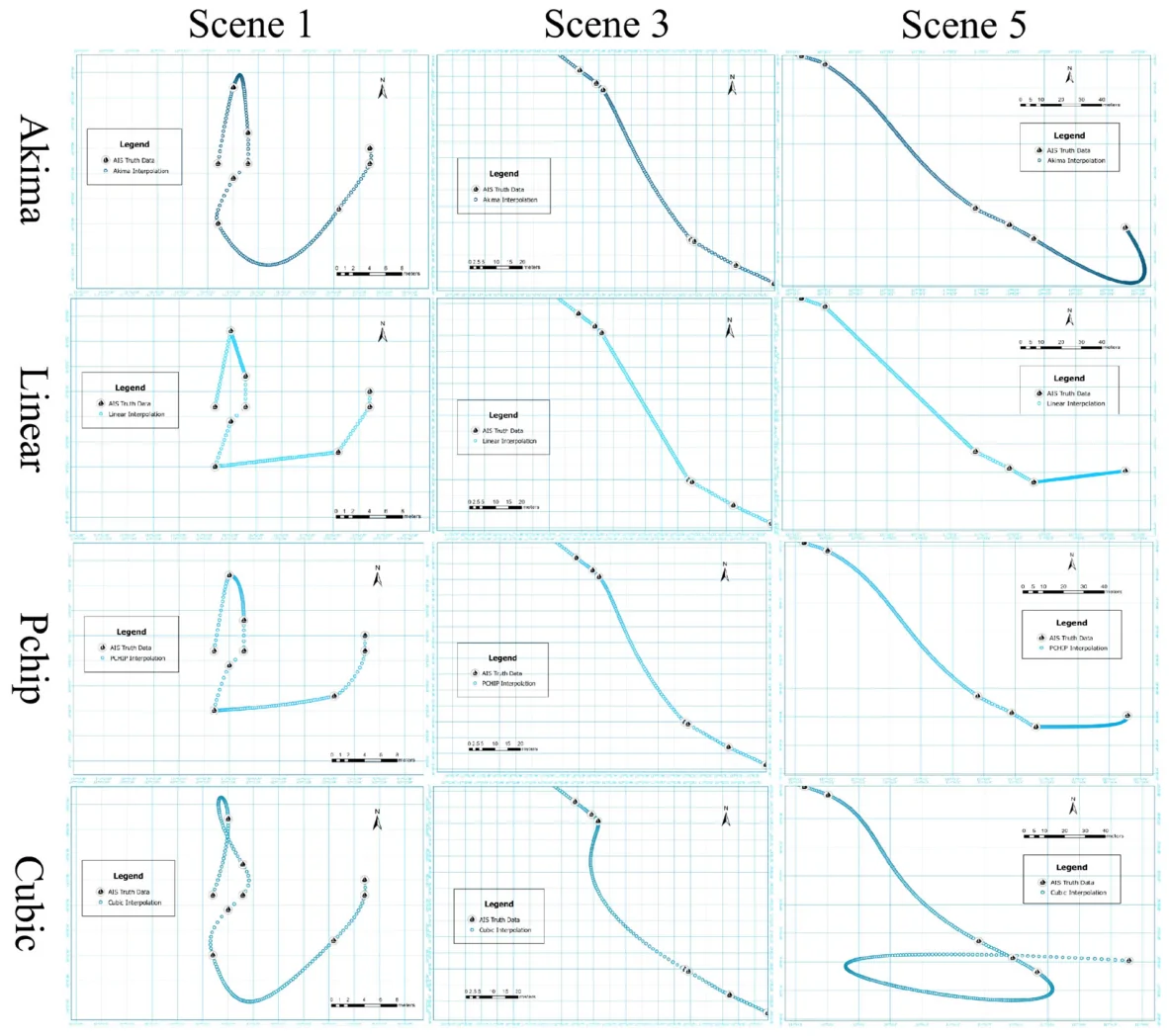
Comparison of AIS trajectory reconstruction algorithm performance. The points in the figure represent original AIS signal points, and the connecting lines denote the interpolation results of each algorithm.

Positional errors were calculated using the Haversine distance, and metrics including MAE, RMSE, Median, P90, and P95 were computed. Here, MAE stands for mean absolute error; RMSE represents root mean square error, which is more sensitive to large errors and reflects the overall error level of the interpolation algorithm; Median, P90, and P95 indicate that 50%, 90%, and 95% of interpolated points have errors not exceeding these values, making them better suited for assessing algorithm stability in engineering applications. The overall trajectory similarity was evaluated using the average DTW (Dynamic Time Warping) distance, which reflects the average closeness between interpolated and true trajectories in terms of overall shape. A total of 318,042 AIS true data points across 383 trajectories were used for accuracy validation in the experiment. The final results of AIS trajectory reconstruction are shown in the Figure 7.

As shown in Table 8, in terms of overall point-level position error, Akima interpolation has the lowest RMSE at 47.91 m, indicating a slight advantage in mean squared error. Moreover, Akima interpolation achieves the best P90 and P95 values among the four methods—18.50 m and 57.53 m respectively—demonstrating a more stable error distribution across most trajectory points. Compared to linear interpolation, Akima interpolation reduces the P95 error by approximately 22.4%, suggesting superior high-percentile error control capability in engineering applications.

The overall trajectory similarity metric further reflects the ability of different algorithms to preserve the general shape of trajectories. As shown in Table 9, Akima interpolation achieves the lowest DTW mean across sampling intervals of 10 s, 30 s, 60 s, 120 s, 300 s, and 600 s, indicating that the interpolated trajectories generated by Akima are closest to the true trajectories in terms of overall shape. This study aims to perform spatiotemporal association between low-precision visual geolocation trajectories and AIS trajectories under complex sea conditions, focusing not only on point-level reconstruction accuracy but also on the overall fidelity of trajectory reproduction. Therefore, Akima interpolation demonstrates its superior performance more distinctly.

### 4.5. Exploring the Weighted Combination of Association Algorithms

The integrated matching cost in this algorithm is determined by position difference, velocity difference, heading difference, and time difference, with corresponding weight parameters W_POS, W_VEL, W_TREND, and W_TIME. From a practical task perspective, AIS-to-visual target matching primarily relies on spatial consistency, so this study focuses on position-dominant parameter configurations—specifically, increasing W_POS while reducing the relative influence of velocity, trend, and time terms. To evaluate the contribution of different motion characteristics to matching performance, the experiments also include parameter combinations such as velocity-dominant, trend-dominant, time-dominant, and uniformly weighted settings. The design aims to analyze the discriminative power of various features in complex maritime multi-target scenarios and identify the feature components that most significantly contribute to matching accuracy. For each weight combination, we evaluate three candidate thresholds corresponding to allowed bandwidth multiples of 1.0, 1.5, and 2.0. Both ISMA and STC-WDTW algorithms adopt the same threshold selection method, choosing the threshold that achieves the best association performance on the training set for subsequent experiments. Both algorithms achieve optimal performance at the 1.5 multiple, so the corresponding threshold value of 0.6753 is fixed in all subsequent weight optimization, ablation, and comparative experiments. All experiments are conducted on the training set, and the test set is not used for threshold selection or exploration of weight combinations. Table 10 presents the parameter settings for the exploratory experiments.

Experimental results show that different parameter combinations significantly affect algorithm performance. The IDP, IDR, IDF1 and MOFA are shown in Table 11.

There are significant differences in the contributions of various features to the AIS-visual target matching task. Specifically, compared with the baseline group, the position-dominant group achieved the best matching performance, with IDF1 increasing to 89.08% and 91.01%, respectively, whereas the speed-dominant group, heading-dominant group, and uniformly weighted group performed relatively worse than the baseline group. This experimental result reflects the applicability characteristics of different features under the current matching framework. First, spatial position is the most critical discriminative feature for this task. Since both AIS data and visual localization results are ultimately converted into a geographic coordinate system, their spatial proximity can directly and comprehensively reflect target correspondence. Second, speed and heading features are constrained by measurement mechanisms and specific scenarios, making them more suitable as fine-grained auxiliary constraints. Taking heading as an example, in typical scenarios such as ports or main shipping lanes, multiple ships often travel densely along similar planned routes, resulting in highly consistent heading values. Therefore, in these specific contexts, relying solely on heading similarity makes it difficult to achieve absolute distinction between different targets. The limitations of the uniform weighting strategy further confirm the necessity of appropriately assigning priority to dominant and secondary features. Thus, in practical applications of AIS-visual target matching algorithms, spatial position should be used as the primary feature, while speed and heading serve as supplementary auxiliary information to enhance discrimination and achieve more robust matching results. Ultimately, this paper recommends a parameter configuration for the dual-strategy matching algorithm with weight ratios of 6:1:1:1 for position, speed, heading, and time features. Subsequent comparative experiments and ablation studies were conducted using the optimal parameters determined in this section.

### 4.6. Association Algorithm Ablation Study

To systematically evaluate the contributions of the spatiotemporal preprocessing pipeline, individual association strategies, and the dual-strategy adaptive switching mechanism “ISMA + STC-WDTW” to the final trajectory association performance, this study follows the principle of controlled variables and conducts six comparative ablation experiments separately on the training and test sets. The experimental results are presented in Table 12 and Table 13.

Experimental results show that the system’s evaluation metrics significantly improve from the baseline group to the complete adaptive dual-strategy model proposed in this paper. Compared with groups ① and ②, the IDF1 scores on both the training and testing sets increase by 15.41 and 11.35 percentage points, respectively, indicating that the underlying spatiotemporal preprocessing pipeline successfully eliminates high-frequency pixel jitter and sampling frequency mismatches, thereby effectively enhancing spatiotemporal consistency. In comparison with single-strategy models, pure ISMA achieves high recall but suffers reduced precision in convergence zones due to its lack of temporal alignment capability, while traditional DTW is limited in recall due to excessive alignment of asymmetric sequences. Comparing groups ④ and ⑤, STC-WDTW improves the IDF1 scores by 4.49 and 6.80 percentage points, respectively, demonstrating its effectiveness in handling complex trajectory associations. Ultimately, group ⑥ achieves IDF1 scores of 93.95% and 93.17% on the training and testing sets and MOFA scores of 88.14% and 86.50%, outperforming all other configurations. Although its precision on the test set is slightly lower than that of group ⑤, it achieves higher recall and overall performance, confirming that the adaptive dual-strategy framework integrates ISMA’s noise robustness in static/simple scenarios with STC-WDTW’s ambiguity resolution capability in highly dynamic/complex convergence scenarios, overcoming the physical applicability limitations of individual algorithms and validating the effectiveness of the proposed method.

### 4.7. Association Algorithm Comparative Experiment

To comprehensively validate the effectiveness of the proposed matching algorithm, this study selects 12 representative algorithms across three categories as control groups, covering classical multi-target association, trajectory similarity measurement, and recent vision-AIS association methods. To ensure a fair comparison, all competing algorithms are reimplemented and run under identical datasets, preprocessing pipelines, and candidate set conditions. Specifically, all methods use the same spatiotemporally consistent reconstructed visual trajectories and AIS trajectories and employ identical spatial and temporal preliminary screening to construct the candidate sets, ensuring completely consistent inputs for each method. The matching results of all methods are compared against the same ground truth annotations, and evaluation metrics are calculated using the same true positive (TP), false positive (FP), and false negative (FN) criteria.

**Figure 8:**
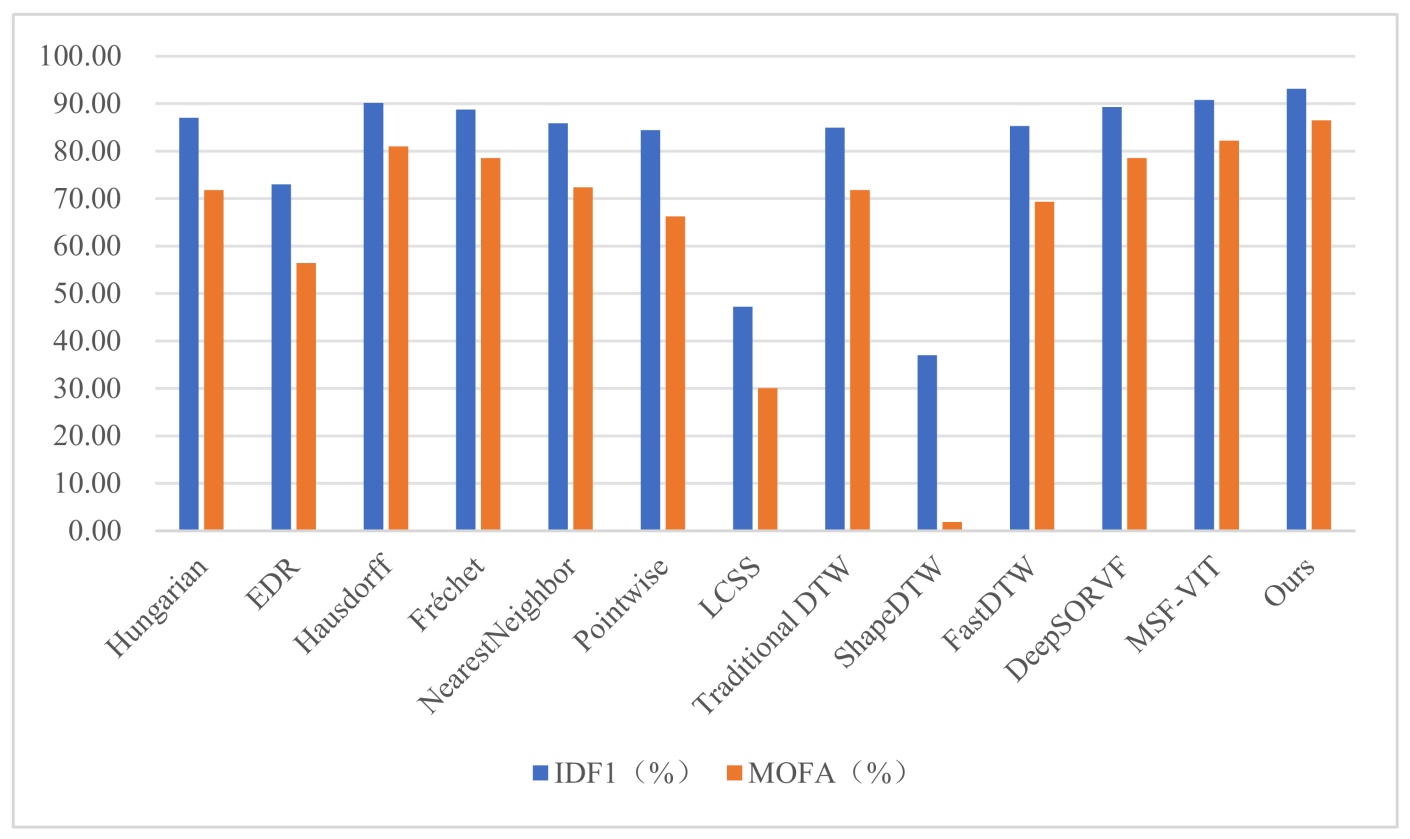
Comparison of IDF1 and MOFA for algorithm association.

**Figure 9:**
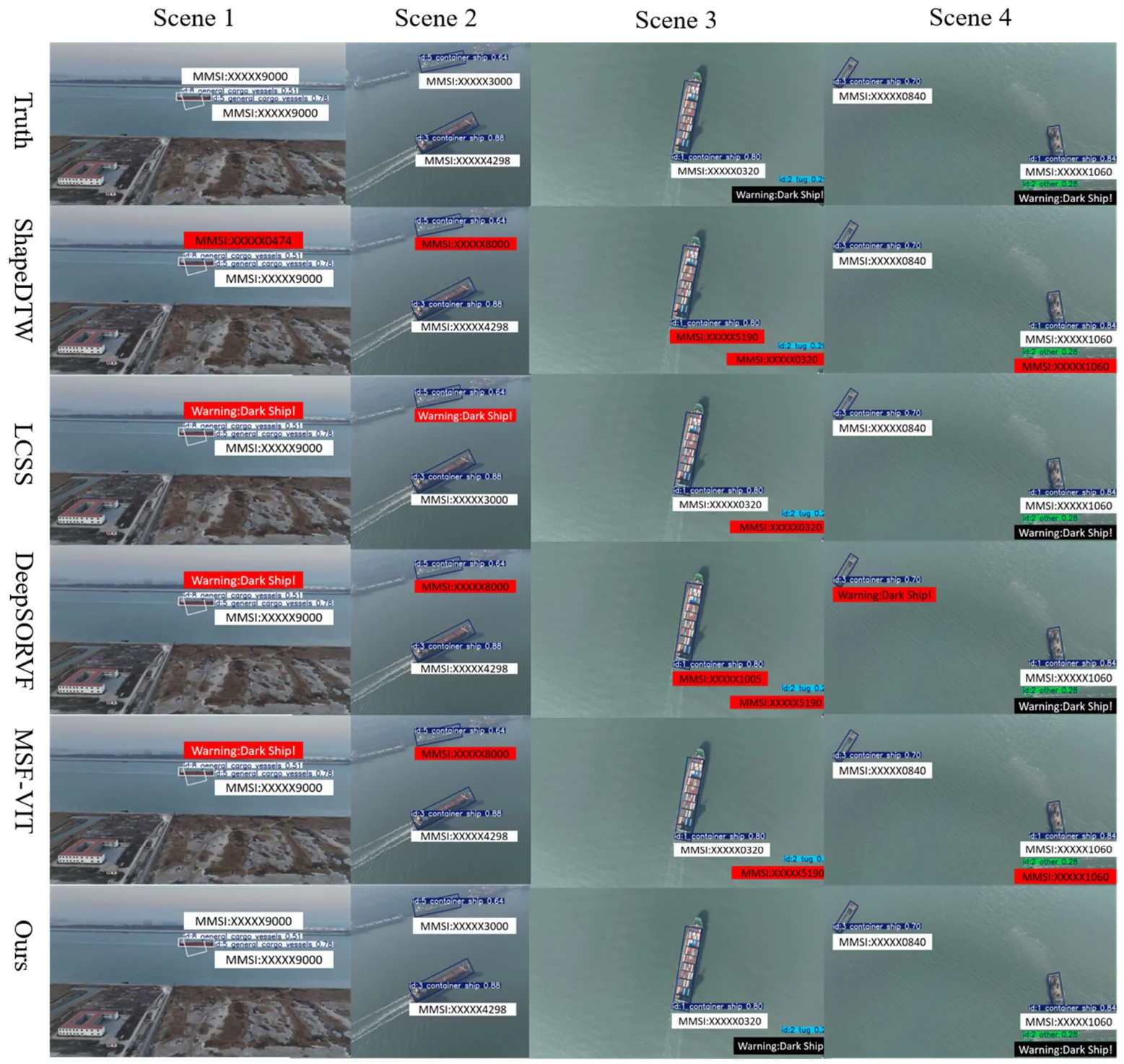
Comparison of partial association algorithm performance. Red labels indicate incorrect matches. In this context, white text on a black background indicates that the vessel should be and is successfully associated; black text on a white background indicates that the vessel should not and is not associated; black text on a red background indicates that the vessel is incorrectly associated; and white text on a red background indicates that the vessel is missed in matching.

In terms of parameter settings, the parameters for each comparison method were selected according to the following principles: NearestNeighbor and Pointwise algorithms directly select the nearest candidate; the Hungarian algorithm does not introduce additional thresholds and instead finds the globally optimal assignment directly on the cost matrix; the matching threshold values for DTW and its variants (including DeepSORVF), as well as distance-based methods such as Fréchet and Hausdorff, along with the matching thresholds for EDR, LCSS, and MSF-VIT (position and heading), were all chosen from the training set to achieve the best F1 score for each method, without using any information from the test set. Our proposed method was compared with all other methods on the same test set, and the results are shown in Table 14 and Figure 8. The performance comparison of several association algorithms is illustrated in Figure 9.

1. Overall Performance Comparison

The proposed method achieves IDF1 and MOFA scores of 93.17% and 86.50%, respectively, both of which are the highest among all compared methods. Compared to the second-ranked MSF-VIT, IDF1 improves by 2.38 percentage points, MOFA by 4.29 percentage points, and IDR also increases by 4.29 percentage points. Although the precision of the proposed method is not the highest, it excels in overall association performance, target coverage capability, and comprehensive evaluation metrics, demonstrating that the proposed approach better balances association accuracy and completeness.

2. Analysis of the classical Euclidean distance matching method

**Figure 10:**
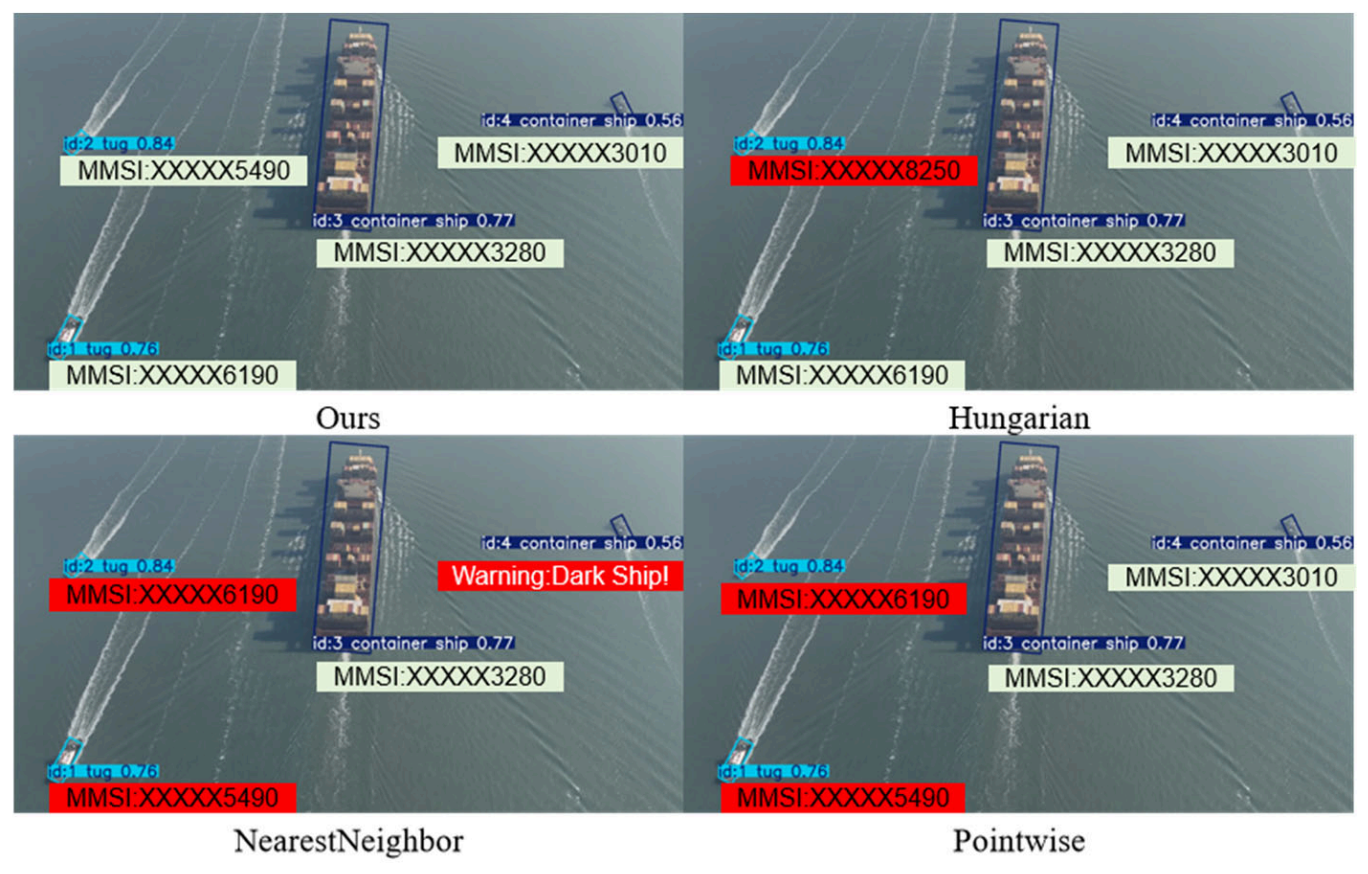
In complex scenarios, the proposed algorithm is compared with the traditional Euclidean distance matching method.

Hungarian and Pointwise achieved IDR scores of 94.48% and 91.41%, respectively, but their IDP scores were only 80.63% and 78.42%. This is because frame-by-frame independent matching lacks global constraints at the trajectory level, making it prone to incorrectly associating spatially adjacent yet actually unrelated targets in multi-object dense scenarios. A common limitation of these two simple methods lies in their lack of modeling for dynamic features and temporal consistency, which makes them inadequate for handling complex multi-object association cases. In contrast, our method outperforms them by 6.16 and 8.75 percentage points in IDF1, respectively. Although NearestNeighbor is simple to implement, its IDF1 score reaches 85.89%, even surpassing that of the DTW algorithm, indicating that matching based on average distance across entire trajectories serves as an effective baseline strategy in visual-AIS matching scenarios. However, its IDF1 is still 7.28 percentage points lower than ours, while MOFA lags by 14.11 percentage points, demonstrating that relying solely on Euclidean distance-based nearest neighbor selection remains insufficient to fully exploit the overall spatiotemporal information within trajectory sequences. The comparison results between the classical Euclidean distance matching method and the proposed method are presented in Table 15 and Figure 10.

3. Analysis of Trajectory Similarity Measurement Methods

Hausdorff and Fréchet show relatively balanced performance, with IDF1 scores of 90.16% and 88.75%, respectively, outperforming traditional DTW and FastDTW but still falling short of the method proposed in this paper. EDR and LCSS exhibit characteristics of “high precision, low recall.” Although they achieve precisions of 96.00% and 96.23%, their recalls are only 58.90% and 31.29%. These two methods rely on Boolean matching strategies and are highly sensitive to distance thresholds, causing many true matches to be filtered due to local deviations. The LCSS feature allowing gaps between trajectories becomes a disadvantage in the current scenario, as there is a significant difference in time coverage between visual and AIS trajectories; excessively long gaps lead to failed matches. ShapeDTW performs the worst, with an IDF1 score of just 37.01%. This is because ShapeDTW normalizes trajectories by translation and scaling and then compares only shape similarity, discarding absolute position information. In the visual-AIS matching context, spatial location is the key criterion for distinguishing different ships; ignoring positional data leads to numerous irrelevant trajectories being incorrectly matched due to similar shapes, resulting in an IDP of only 51.65%. This result further confirms the critical role of spatial position information in this task.

4. Analysis of Recent Visual-AIS Association Methods

DeepSORVF and MSF-VIT represent state-of-the-art approaches in current visual-AIS association. However, it should be noted that, like this paper, both algorithms employ deep learning methods only in the ship detection and tracking stages, while still relying on rule-based methods for matching AIS data with visual targets. DeepSORVF performs association using an enhanced fast dynamic time warping (E-FastDTW) algorithm after extracting pixel trajectories from both visual and AIS data, achieving moderate performance with a precision of 89.02%, recall of 89.57%, and IDF1 score of 89.30%. MSF-VIT achieves multi-constraint matching by extracting AIS pixel-domain coordinates and the actual heading of visual targets, ranking second among all compared methods.

## 5. Discussion

### 5.1. Quantitative Refinement of Monocular Projection Error and Conditional Boundaries for Drones

Previous research on visual-AIS spatial alignment has primarily focused on constructing the overall transformation pipeline or has often been conducted under ideal assumptions (such as assuming absolute drone stability or neglecting distortion amplification at small angles). This study further quantifies the coupled effects of gimbal pitch angle, local observation angle, and slant range through controlled variable analysis:

1. Cloud platform pitch angle (the angle between the camera optical axis and the aircraft’s horizontal reference plane).

**Figure 11:**
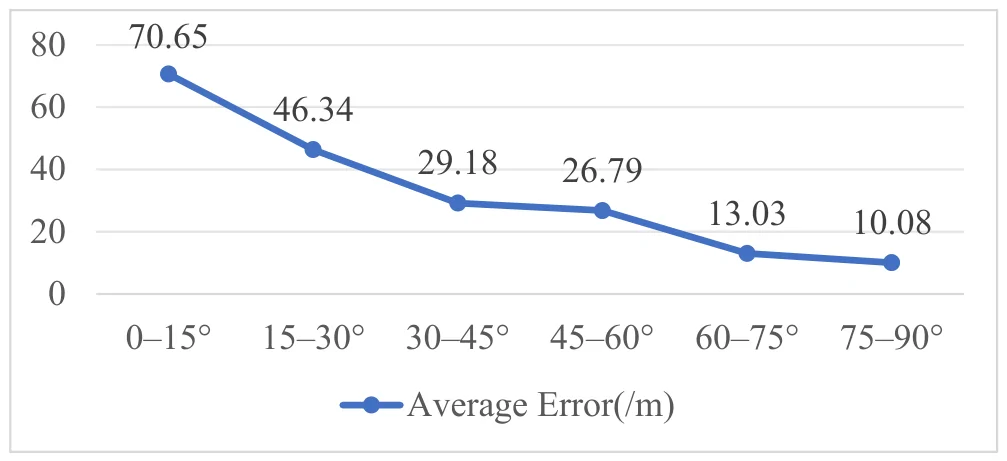
Analysis of Positioning Error Factors I.

As shown in Figure 11, the experimental results indicate that the gimbal pitch angle primarily affects the underlying baseline accuracy by altering the degree of perspective compression across the full frame. Although low-altitude, sea-level viewing angles provide rich side textures [58], this study finds that when the pitch angle is small (15–30°), the micro-frequency vibrations of the onboard inertial navigation system are globally amplified under perspective projection, causing positioning deviations. As the gimbal pitch angle increases gradually (approaching 60–90° vertical downward view), the camera’s optical axis becomes nearly perpendicular to the sea surface, resulting in reduced geometric projection distortion across the entire image and suppressing projection errors caused by static residuals from attitude sensors.

2. The influence of the angle between the line connecting the target and the drone’s camera and the horizontal plane (denoted as α).

**Figure 12:**
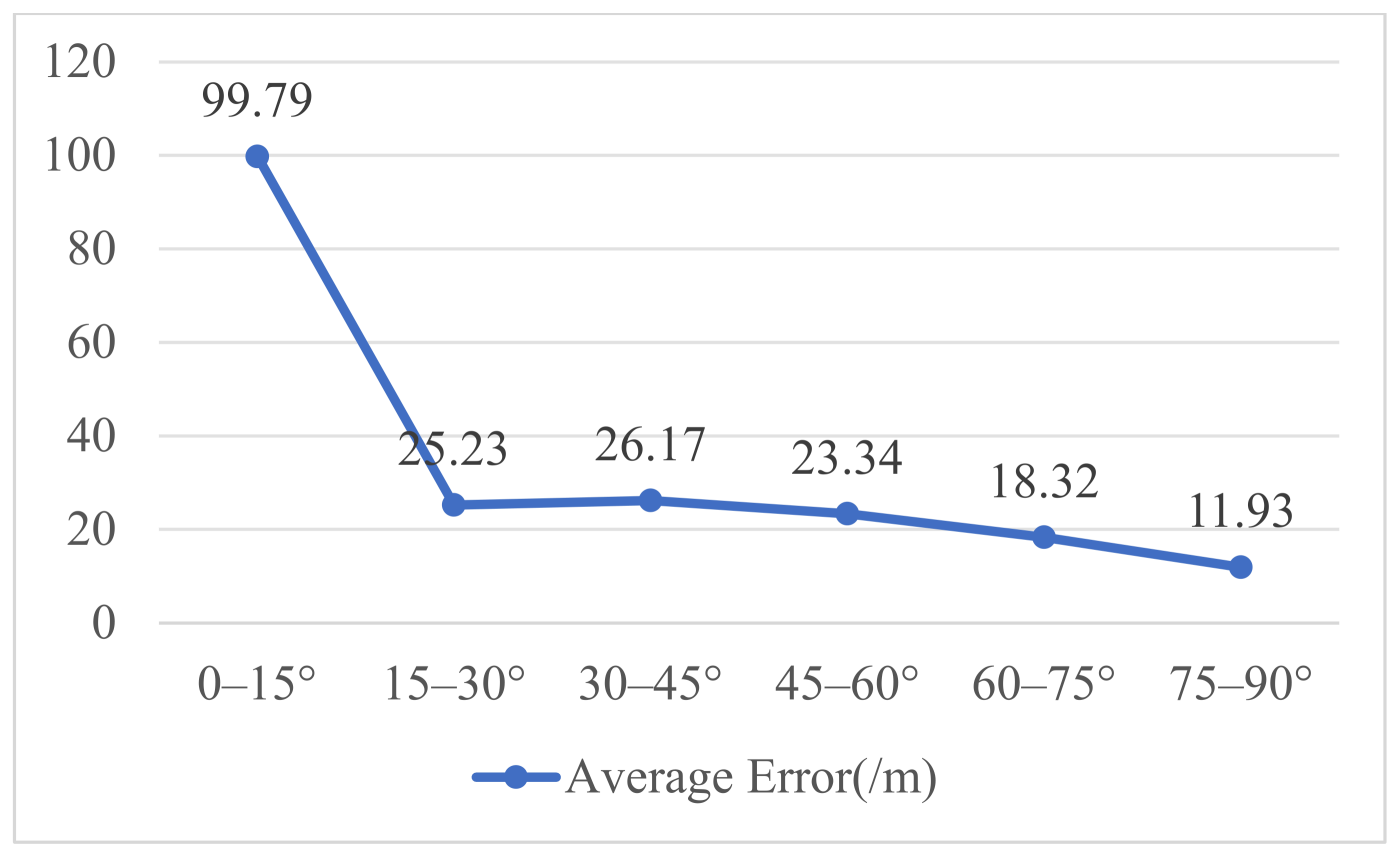
Analysis of Positioning Error Factors II.

Unlike the global reference pitch angle of the gimbal, the angle between the line connecting the target and the drone’s camera lens and the horizontal plane is a local observation angle. Since the position of the target ship on the image plane changes dynamically, this angle equals the gimbal pitch angle only when the target is precisely located at the principal point in the center of the image. Its relationship with positioning accuracy is shown in Figure 12.

According to the inverse geodetic coordinate model, the ground projection distance is inversely proportional to tan(*α*). Differentiating this relationship shows that the rate of change in spatial positioning error with respect to angle is directly proportional to $\frac{1}{\sin^{2}(\alpha )}$. Therefore, when the target is located near the top edge of the video, resulting in a very small angle *α*, even minor disturbances—such as ship body oscillations caused by waves or edge jitter of just 1–2 pixels introduced by object detection algorithms—are nonlinearly amplified and can translate into drift errors of several meters or even tens of meters in the geodetic coordinate system.

3. The Impact of Distance Between the Target and the Drone Camera.

**Figure 13:**
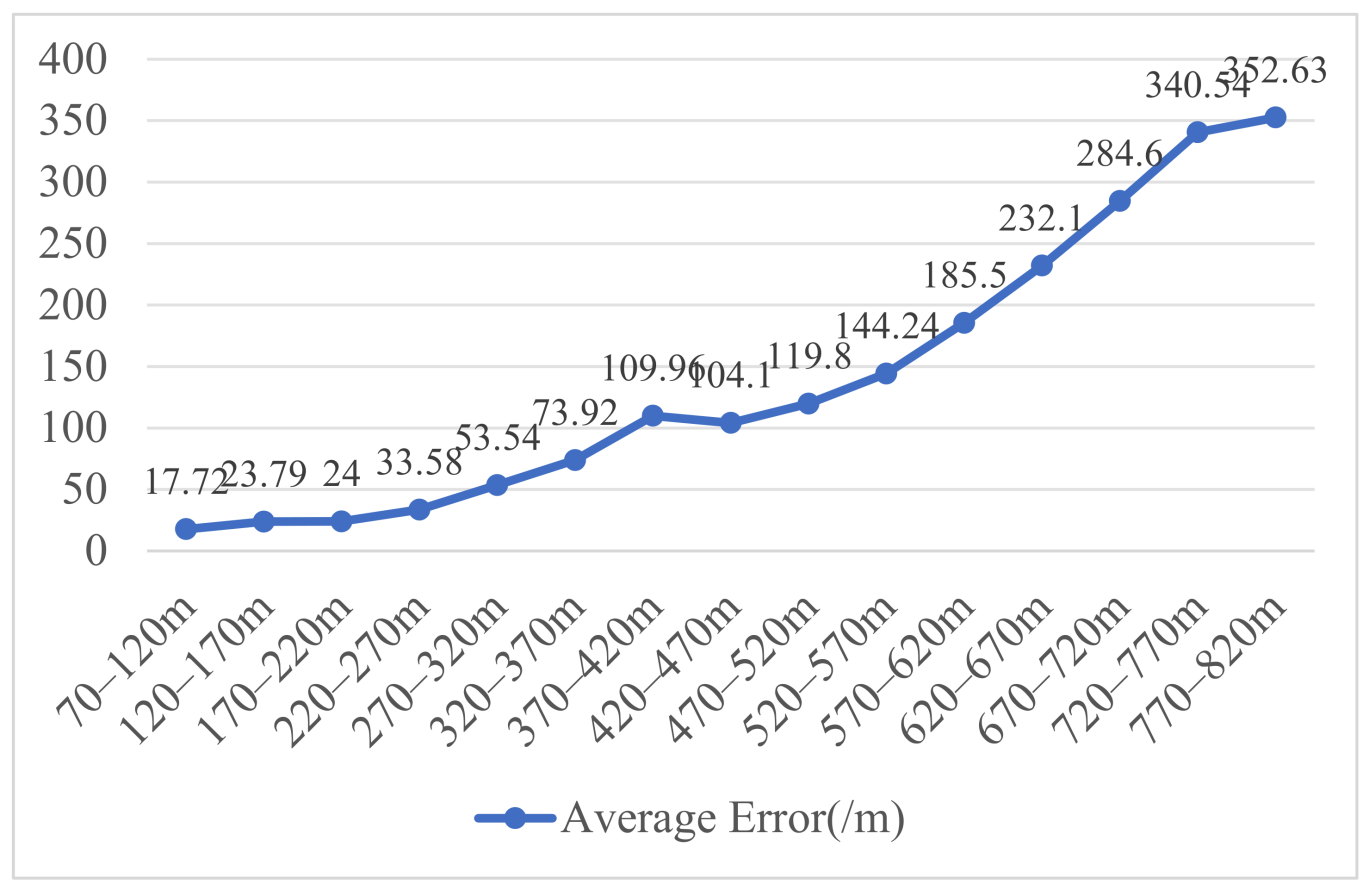
Analysis of Positioning Error Factors III.

As shown in Figure 13, experimental data confirm that as the distance increases from 50 m to over 500 m, the degradation of ground sample distance (GSD) combines with the amplification effect of angular errors, leading to a sharp decline in positioning accuracy. Therefore, observation distance not only affects image resolution and target detection accuracy but also limits the precision of monocular visual inverse positioning, confirming that distance is a critical factor constraining the accuracy of monocular visual inverse calculation [59,60].

### 5.2. Performance Comparison of Association Algorithms

In high-dynamic drone maritime monitoring scenarios, spatiotemporal association of multi-source heterogeneous data imposes stringent requirements on algorithm robustness. Based on the results of ablation and comparative experiments, the proposed “ISMA + STC-WDTW” adaptive dual-strategy association framework demonstrates significant advantages in two dimensions:

1. Under the premise of the objective existence of systematic errors, scene-driven adaptive high-tolerance association has been achieved.

Although traditional point-by-point matching is sensitive to local trajectories, it is prone to false matches in areas with multiple vessels operating in parallel or crossing paths. On the other hand, strict distance constraints (such as nearest neighbor NN) lead to numerous missed matches due to overly conservative strategies. While conventional DTW algorithms introduce temporal alignment, they are highly susceptible to “over-smoothing” when dealing with drones’ short-term fragmented visual trajectories and AIS sequences with intermittent data gaps. The proposed STC-WDTW algorithm fundamentally overcomes matching deadlocks caused by spatiotemporal asynchrony and data truncation by incorporating multidimensional motion feature verification, adaptive time penalties, and open start/end boundaries.

Furthermore, ablation experiments reveal the true feature weight distribution in heterogeneous associations: spatial position features mapped to a unified geographic coordinate system dominate the discrimination, while velocity and heading serve as refined auxiliary constraints.

2. By utilizing physically observable features, the reliance on non-positional attributes of AIS is reduced.

Existing visual-AIS fusion studies often implicitly assume that non-position attributes broadcast by AIS, such as speed over ground (SOG) and course over ground (COG), are absolutely accurate. However, in practical applications, these broadcast values may be distorted or exhibit sudden jumps due to system errors or human factors. This paper’s dual-strategy algorithm reduces direct reliance on AIS non-position attributes; the required velocity and heading features for matching are instead derived by back-calculating from trajectory points reconstructed in physical space through visual tracking. This feature-based extraction mechanism not only ensures consistency in spatiotemporal characteristics across domains but also effectively prevents association failures caused by errors in directly reading AIS data. Moreover, the proposed method holds potential application value in identifying anomalous AIS information.

### 5.3. Limitations of the Proposed Method

Although this paper has made systematic optimizations in the underlying spatiotemporal alignment and upper-level association architecture, due to objective physical constraints and existing technological limitations, the study still faces certain limitations in practical applications:

1. The physical limitations of drone video positioning constrain the effective regulatory radius.

**Figure 14:**
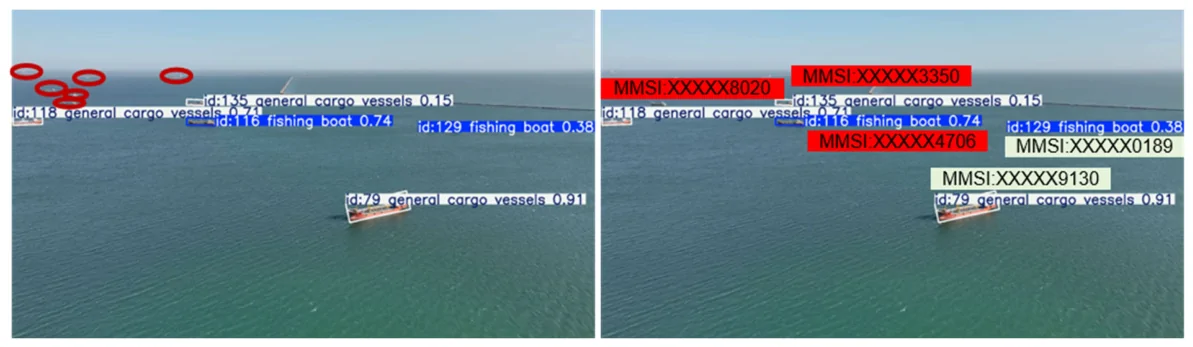
Algorithm accuracy decreases due to insufficient observation conditions.

As discussed in this paper’s investigation of positioning errors, the target localization method employed here is essentially a geodetic coordinate back-calculation approach based on monocular visual projection models. Its positioning accuracy is objectively constrained by imaging geometry and sensor error propagation characteristics. Experimental results show that factors such as gimbal pitch angle, target observation angle, and target distance significantly affect positioning accuracy. The proposed method recommends an observation distance of 300 m and a pitch angle of 60°; the maximum applicable observation distance is 500 m with a maximum pitch angle of 45°, beyond which the accuracy of association algorithms will be significantly compromised. As shown in the video data in Figure 14, which exceeds 10 min in duration and contains numerous ships within the field of view, the corresponding drone pitch angles range between 5° and 15°, with a maximum observation distance exceeding 1 km, resulting in a significant degradation in target detection, tracking, and monocular visual localization accuracy.

2. Time synchronization of the underlying hardware and optical distortion still involve uncontrollable errors.

Unlike surveying cameras equipped with mechanical shutter synchronization mechanisms, conventional drone video streams and SRT metadata exhibit millisecond-level latency fluctuations during software loading and transmission. Additionally, the lack of fixed camera intrinsic parameters for real-time dynamic stabilization and default distortion correction makes it difficult to accurately reverse-calculate actual image distortions. Although this paper employs time-axis linear interpolation and state smoothing, these systematic parameter deviations still define the practical limits for high-precision real-time monitoring using drone video.

3. Temporal instability of visual perception models under complex sea conditions.

In real-world sea surface monitoring, drastic changes in lighting, sea clutter, and strong ship wake interference make it difficult for target detection algorithms (such as YOLO) to maintain absolutely stable feature extraction. The edges of bounding boxes (BBox) are prone to random shrinking, expansion, or rotational jumps across consecutive frames, causing the localization anchors to oscillate locally in the pixel domain. Although introducing Kalman filtering can effectively smooth trajectories, this underlying distortion arising from visual perception uncertainty still carries a risk of local false matches under extreme sea conditions.

### 5.4. Future Outlook

In response to the objective limitations revealed in current research, future work will primarily focus on intensive efforts in the following three directions:

1. Break through the physical distance measurement limit of monocular vision and expand the system’s effective monitoring radius.

To address the issue of random coordinate drift caused by GSD degradation in monocular cameras at long distances, future research could explore integrating multimodal onboard sensors—such as lightweight laser rangefinders or stereo vision cameras—to provide depth constraints for geographic projection models. Meanwhile, on the algorithmic side, deep learning-based monocular depth estimation networks could be introduced to leverage geometric priors from sea surface scenes, mitigating nonlinear amplification effects at large slant ranges. This approach would help overcome the current physical limitations of monocular visual monitoring and enable high-precision positioning beyond line-of-sight.

2. Establish a fine-grained error compensation mechanism integrating software and hardware to strengthen the underlying spatiotemporal reference framework.

To address the millisecond-level software loading delays and dynamic distortion issues between conventional drone video streams and SRT metadata, future maritime-specific airborne systems should evolve toward deep integration of hardware and software. On the one hand, research should explore a microsecond-level camera-IMU (inertial measurement unit) timing synchronization mechanism based on hardware pulse triggering to completely eliminate temporal misalignment. On the other hand, real-time adaptive calibration algorithms for camera intrinsic parameters should be developed to account for drone video dynamic stabilization characteristics, thereby eliminating systematic residuals in geopositional projection calculations at the source of hardware measurements and imaging.

3. Introduce a vision perception model with temporal joint feature optimization to enhance the robustness of front-end tracking.

To address the issues of temporal fluctuations in detection bounding boxes and OBB jumps under complex sea conditions (such as abrupt lighting changes and long wakes) and specific viewing angles, future research should upgrade visual perception frameworks from the current “frame-independent detection” to “temporal joint detection.” By embedding optical flow features or spatiotemporal memory modules (e.g., ConvLSTM) into object detection neural networks, contextual information from adjacent frames can be leveraged to directly suppress abnormal shrinkage and discontinuous rotation of local bounding boxes at the network level.

## 6. Conclusions

To address the practical challenges in maritime surveillance—such as short-term fragmentation of visual trajectories caused by high-dynamic drone observations and difficulties in accurately associating these with low-frequency AIS trajectories across spatiotemporal dimensions—this paper proposes a trajectory association method based on strategy switching driven by motion salience and scene complexity. Technically, the approach employs monocular geolocation, Kalman smoothing, and Akima interpolation under kinematic constraints as data cleaning techniques to achieve spatiotemporal alignment between visual and AIS trajectories in geographic space. Considering the characteristics of open-boundary observation from high-dynamic drone platforms and low-frequency AIS data, this study develops a dual-strategy adaptive association mechanism. It applies ISMA in low-complexity/high-salience scenarios and automatically switches to STC-WDTW when facing complex spatiotemporal constraints or cases involving trajectory offset or truncation. This mechanism effectively overcomes the limitations of traditional Euclidean distance matching, enabling robust association of asynchronous and fragmented trajectories. Experimental results demonstrate that the method can effectively handle real-world challenges such as drone recording interruptions, broken visual trajectories, and AIS data asynchrony. Based on the association outcomes, the method also holds potential research value for detecting AIS anomalies, including dark ships failing to report and speed/course spoofing. This study provides technical support and engineering significance for intelligent, proactive maritime surveillance under high-dynamic observational conditions.

## Figures and Tables

**Table 1:** Main findings and limitations of the current study.

Research Areas	Key Findings	Limitations
Aerial and maritime multimodal perception	Complementary information can effectively improve perception accuracy [21].	AIS relies on cooperative objectives and has a low update rate; it faces challenges related to asynchronicity [46].
Visual-AIS Spatial Alignment	Implement the conversion pipeline between pixel coordinates and geographic coordinates [22].	Onboard georeferencing is difficult; monocular video projection has large errors [23].
Heterogeneous Data Time Synchronization	Employing strategies such as interpolation, linear prediction, and motion perception [17,28].	Depends on calibration accuracy, data synchronization quality, and association logic [17,41].
Classic trajectory association algorithm	Introduce multidimensional feature comparisons of trajectory shapes to reduce association ambiguity [41].	Unable to robustly match under high-frequency noise and varying scenarios [41].
Deep learning association algorithms	Obtain spatiotemporal interactive features of ship trajectories to achieve asynchronous matching [44,45,46].	Lack of airborne paired datasets with spatiotemporally synchronized annotations; interpretability issues [28].

**Table 2:** Feature normalization parameters.

Sensor Accuracy Parameters	Value	Base
Position bandwidth *σ_pos_*	100	Initial field tests show that the monocular drone positioning error is approximately 100 m at a slant range of 500 m.
Speed bandwidth *σ_vel_*	5	The initial field test showed a speed measurement error of approximately 5 m/s.
Course bandwidth *σ_course_*	25	Initial field measurements indicate a heading error of approximately 25°.
Time-bandwidth *σ_time_*	3	Maximum time window: 3 s.

**Table 3:** Candidate composite cost thresholds.

Tolerance Level D*_th_*	Quadratic Form Threshold D_th_^2^	Composite Cost Threshold C*_th_*	Meaning
1.0	1.00	0.3935	The root mean square of weighted standardized residuals does not exceed 1 bandwidth scale.
1.5	2.25	0.6753	The root mean square of the weighted standardized residuals does not exceed 1.5 bandwidth scales.
2.0	4.00	0.8647	The root mean square of weighted standardized residuals does not exceed 2 bandwidth scales.

**Table 4:** Field Data Summary.

Project	Numerical Value
Drone platform	DJI M3E and DJI M4E
AIS receiver	HS-1206 and HS-6206GT
Video sequence	146 paragraphs
Effective video duration	511 min
Average duration per segment	3.5 min
Effective AIS record after cleaning	56,338 entries
Vessels with independent identity tags	370 ships
Observation distance	70–800 m
Observation geometry range	Approximately 5–90°
Collection site	Port waters and offshore work zones
Flight Level Stratification	<30 m, 30–100 m, >100 m
Scene Type	Single vessel, parallel, opposing, crossing, anchored
Environmental lighting conditions	Dawn, noon, dusk

**Table 5:** Geodetic Coordinate Inversion Accuracy Test Scenario I.

Pitch	Mean (/m)	Min (/m)	Max (/m)
0–15°	8.36	1.61	18.83
15–30°	12.99	0.77	48.00
30–45°	6.50	2.48	14.57
45–60°	7.37	1.04	17.63
60–75°	12.12	10.15	16.23
75–90°	14.95	7.13	21.43

**Table 6:** Geodetic Coordinate Inversion Accuracy Test Scenario II.

Pitch	Mean (/m)	Min (/m)	Max (/m)
0–15°	70.65	4.07	355.95
15–30°	46.34	2.05	158.47
30–45°	29.18	2.82	68.09
45–60°	26.79	13.72	45.53
60–75°	13.03	2.35	25.06
75–90°	3.66	1.55	5.44

**Table 7:** Geodetic Coordinate Inversion Accuracy Test Scenario III.

Pitch	Mean (/m)	Min (/m)	Max (/m)
0–15°	6.03	3.16	7.73
15–30°	4.03	2.42	7.40
30–45°	4.07	1.25	6.50
45–60°	3.67	1.25	6.59
60–75°	3.87	2.78	6.21
75–90°	4.54	1.64	9.18

**Table 8:** Overall Comparison of Interpolation Position Accuracy. The best values are highlighted in bold.

Algorithm	MAE/m	RMSE/m	Median/m	P90/m	P95/m
Akima	**11.84**	**47.91**	0.55	**18.5**	**57.53**
Cubic	**11.84**	49.09	0.58	20.51	58.61
Pchip	12.36	49.66	**0.54**	20.91	62.24
Linear	14.47	57.73	0.57	24.31	74.18

**Table 9:** Comparison of overall trajectory similarity (DTW mean/m). The best values are highlighted in bold.

Sampling Interval (s)	Akima	Cubic	Pchip	Linear
10	**0.59**	0.62	0.60	0.61
30	**0.85**	0.86	0.89	0.96
60	**1.33**	1.38	1.52	1.75
120	**2.49**	2.60	2.80	3.24
300	**5.92**	6.05	6.09	6.96
600	**13.51**	14.18	13.91	14.95

**Table 10:** Parameter Combinations.

Group	Strategy	W_POS	W_VEL	W_TREND	W_TIME
G01	ISMA	1.5	0.5	0.5	\
G02	ISMA	3.0	0.5	0.5	\
G03	ISMA	0.5	3.0	0.5	\
G04	ISMA	0.5	0.5	3.0	\
G05	ISMA	1.0	1.0	1.0	\
G06	STC-WDTW	1.5	0.5	0.5	0.5
G07	STC-WDTW	3.0	0.5	0.5	0.5
G08	STC-WDTW	0.5	3.0	0.5	0.5
G09	STC-WDTW	0.5	0.5	3.0	0.5
G10	STC-WDTW	0.5	0.5	0.5	3.0
G11	STC-WDTW	1.0	1.0	1.0	1.0

**Table 11:** Experimental Results of Optimal Parameter Investigation. The optimal parameter combinations for the two algorithms are highlighted in bold.

Group	IDP (%)	IDR (%)	IDF1 (%)	MOFA (%)
G01	94.57	49.15	64.68	46.33
**G02**	**88.33**	**89.83**	**89.08**	**77.97**
G03	57.45	45.76	50.94	11.86
G04	51.64	35.59	42.14	2.26
G05	91.82	57.06	70.38	51.98
G06	93.84	77.40	84.83	72.32
**G07**	**93.45**	**88.70**	**91.01**	**82.49**
G08	71.43	64.97	68.05	38.98
G09	90.24	41.81	57.14	37.29
G10	62.61	40.68	49.32	16.38
G11	93.08	68.36	78.83	63.28

**Table 12:** Ablation Experiment Results I—Training Set. The best values are highlighted in bold.

Configuration	IDP (%)	IDR (%)	IDF1 (%)	MOFA (%)
① Nearest neighbor without smoothing/interpolation	85.50	63.28	72.73	52.54
② Use smooth and Akima’s NN	88.14	88.14	88.14	76.27
③ Only ISMA	88.33	89.83	89.08	77.97
④ Traditional DTW	86.03	87.01	86.52	72.88
⑤ Only STC-WDTW	93.45	88.70	91.01	82.49
⑥ Adaptive ISMA+STC-WDTW	**95.88**	**92.09**	**93.95**	**88.14**

**Table 13:** Ablation Experiment Results II—Test Set. The best values are highlighted in bold.

Configuration	IDP (%)	IDR (%)	IDF1 (%)	MOFA (%)
① Nearest neighbor without smoothing/interpolation	93.52	61.96	74.54	57.67
② Use smooth and Akima’s NN	87.82	84.05	85.89	72.39
③ Only ISMA	92.67	85.28	88.82	78.53
④ Traditional DTW	90.91	79.75	84.97	71.78
⑤ Only STC-WDTW	**94.77**	88.96	91.77	84.05
⑥ Adaptive ISMA+STC-WDTW	94.34	**92.02**	**93.17**	**86.50**

**Table 14:** Performance comparison of association methods. The best values are highlighted in bold.

Algorithm	IDP (%)	IDR (%)	IDF1 (%)	MOFA (%)
Hungarian	80.63	**94.48**	87.01	71.78
EDR	96.00	58.90	73.00	56.44
Hausdorff	93.42	87.12	90.16	80.98
Fréchet	93.24	84.66	88.75	78.53
NearestNeighbor	87.82	84.05	85.89	72.39
Pointwise	78.42	91.41	84.42	66.26
LCSS	**96.23**	31.29	47.22	30.06
TraditionalDTW	90.91	79.75	84.97	71.78
ShapeDTW	51.65	28.83	37.01	1.84
FastDTW	81.92	88.96	85.29	69.33
DeepSORVF	89.02	89.57	89.30	78.53
MSF-VIT	94.08	87.73	90.79	82.21
**Ours**	94.34	92.02	**93.17**	**86.50**

**Table 15:** The proposed method achieves superior performance metrics compared with the classical Euclidean distance matching approach. ↑ denotes the extent to which the proposed method outperforms this method, while ↓ denotes the extent to which the proposed method underperforms this method.

Index	Ours	Compare with Hungarian	Compare with NearestNeighbor	Compare with Pointwise
IDP (%)	94.34	↑ 13.71	↑ 6.52	↑ 15.92
IDR (%)	92.02	↓ 2.45	↑ 7.98	↑ 0.61
IDF1 (%)	93.17	↑ 6.16	↑ 7.27	↑ 8.75
MOFA (%)	86.50	↑ 14.72	↑ 14.11	↑ 20.25

## Data Availability

The datasets and code are available from the corresponding author upon reasonable request.
